# Basic newborn care and neonatal resuscitation: a multi-country analysis of health system bottlenecks and potential solutions

**DOI:** 10.1186/1471-2393-15-S2-S4

**Published:** 2015-09-11

**Authors:** Christabel Enweronu-Laryea, Kim E Dickson, Sarah G Moxon, Aline Simen-Kapeu, Christabel Nyange, Susan Niermeyer, France Bégin, Howard L Sobel, Anne CC Lee, Severin Ritter von Xylander, Joy E Lawn

**Affiliations:** 1 Department of Child Health, School of Medicine and Dentistry, College of Health Sciences University of Ghana, Accra, PO Box 4236, Ghana; 2 Health Section, Programme Division, UNICEF Headquarters, 3 United Nations Plaza, New York, NY 10017, USA; 3 Maternal, Adolescent, Reproductive and Child Health (MARCH) Centre, London School of Hygiene and Tropical Medicine, London, WC1E 7HT, UK; 4 Saving Newborn Lives, Save the Children, 2000 L Street NW, Suite 500, Washington, DC 20036, USA; 5 Department of Infectious Disease Epidemiology, London School of Hygiene and Tropical Medicine, London, WC1E 7HT, UK; 6 Ross University Medical School, 2300 SW 145th Avenue, Miramar, Florida 33027, USA; 7 Section of Neonatology, University of Colorado School of Medicine, 13121 E. 17th Avenue, Aurora, CO 80045, USA; 8 IYCN, UNICEF Headquarters, 3 United Nations Plaza, New York, NY 10017, USA; 9 Reproductive, Maternal, Newborn, Child and Adolescent Health, Division of NCD and Health through Life-Course, World Health Organization, Regional Office for the Western Pacific, Manila, Philippines; 10 Department of Pediatric Newborn Medicine, Brigham and Women's Hospital, 75 Francis Street, Boston, MA 02115, USA; 11 Department of Maternal, Newborn, Child and Adolescent Health, World Health Organization, 20 Avenue Appia, 1211 Geneva 27, Switzerland

**Keywords:** Quality of care, bottleneck analysis, Africa, Asia, health system, resuscitation, basic newborn care

## Abstract

**Background:**

An estimated two-thirds of the world's 2.7 million newborn deaths could be prevented with quality care at birth and during the postnatal period. Basic Newborn Care (BNC) is part of the solution and includes hygienic birth and newborn care practices including cord care, thermal care, and early and exclusive breastfeeding. Timely provision of resuscitation if needed is also critical to newborn survival. This paper describes health system barriers to BNC and neonatal resuscitation and proposes solutions to scale up evidence-based strategies.

**Methods:**

The maternal and newborn bottleneck analysis tool was applied by 12 countries in Africa and Asia as part of the Every Newborn Action Plan process. Country workshops engaged technical experts to complete the survey tool, which is designed to synthesise and grade health system "bottlenecks" that hinder the scale up of maternal-newborn intervention packages. We used quantitative and qualitative methods to analyse the bottleneck data, combined with literature review, to present priority bottlenecks and actions relevant to different health system building blocks for BNC and neonatal resuscitation.

**Results:**

Eleven of the 12 countries provided grading data. Overall, bottlenecks were graded more severely for resuscitation. The most severely graded bottlenecks for BNC were health workforce (8 of 11 countries), health financing (9 out of 11) and service delivery (7 out of 9); and for neonatal resuscitation, workforce (9 out of 10), essential commodities (9 out of 10) and service delivery (8 out of 10). Country teams from Africa graded bottlenecks overall more severely. Improving workforce performance, availability of essential commodities, and well-integrated health service delivery were the key solutions proposed.

**Conclusions:**

BNC was perceived to have the least health system challenges among the seven maternal and newborn intervention packages assessed. Although neonatal resuscitation bottlenecks were graded more severe than for BNC, similarities particularly in the workforce and service delivery building blocks highlight the inextricable link between the two interventions and the need to equip birth attendants with requisite skills and commodities to assess and care for every newborn. Solutions highlighted by country teams include ensuring more investment to improve workforce performance and distribution, especially numbers of skilled birth attendants, incentives for placement in challenging settings, and skills-based training particularly for neonatal resuscitation.

## Background

Substantial reductions in childhood deaths have occurred since 1990, but deaths in the first 28 days of life (newborn period) have declined more slowly [1]. Globally, newborn deaths accounted for an estimated 44% of deaths in children under the age of 5 years in 2013 [2,3]. Sub-Saharan African region now has the greatest neonatal mortality burden as the trend in South Asia shows relatively faster decline. Intrapartum-related hypoxia, infections and prematurity or small size at birth account for 85% of newborn deaths globally [4]. One million of the 2.7 million neonatal deaths in 2013 occurred on the first day of life -the most critical period for survival and prevention of long-term disability [1,4-6]. A significant proportion of fresh stillbirths and deaths on the first day are caused by intrapartum-related complications. As many as two-thirds of newborns deaths could be averted with quality care at birth and during the postpartum period [4,5,7].

**Figure 1:**
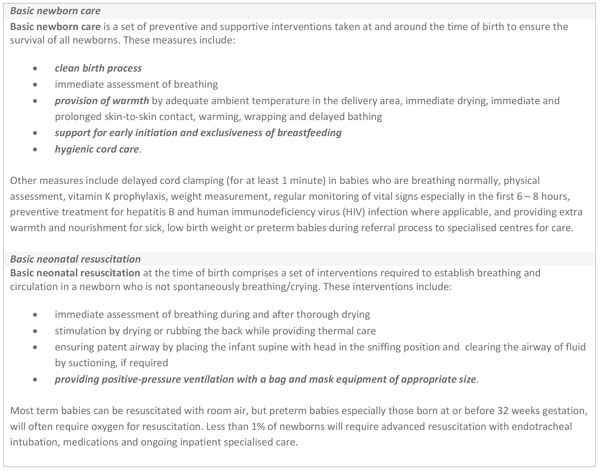
**Definitions and tracer interventions**. Texts in italics are tracer interventions selected for the bottleneck analysis.

Basic newborn care (BNC) comprises of a set of basic preventive and supportive measures that are needed to ensure the survival, health and development of all newborns (Figure 1). For the purpose of this analysis, BNC focuses especially on cleanliness and cord care, thermal control (including drying, skin-to-skin care, delayed bathing), and support for breastfeeding. In addition, the paper considers neonatal resuscitation - a set of interventions to support the establishment of breathing and circulation for babies who require assistance to breathe at birth [8] - of which the most important is positive pressure ventilation with bag and mask, which is also the focus of this analysis (Figure 1). These low-cost procedures could prevent many newborn deaths around the time of birth and on the first day of life.

Providing quality intrapartum care and resuscitation is a vital opportunity to ensure a good start in life for all newborns, and skilled birth attendants hold the key to quality survival of every newborn. Clean birth processes reduce the risk of intrapartum-acquired neonatal infections [9,10]; and provision of external source of warmth, including skin-to-skin, enables normal transition from fetal life and reduces deaths from hypothermia [11,12]. Early initiation of breastfeeding in the first hour of life is associated with a lower risk of neonatal mortality [13]. Hygienic newborn practices including cord care prevents postnatal infections [14], a major cause of neonatal deaths, and in specific settings the application of antiseptics to the cord have additional benefit [14,15]. Skilled care and assessment during the early postnatal period, especially on the first day of life provides an opportunity to detect and manage complications that may result in early neonatal death [4,16]. Providing resuscitation, when needed, could avert approximately 30% of term newborn deaths and 10% of preterm deaths in the first month of life [17]. Nevertheless, these low-cost quality services are not being implemented at scale in settings where they are likely to have the most impact [18-20].

**Figure 2:**
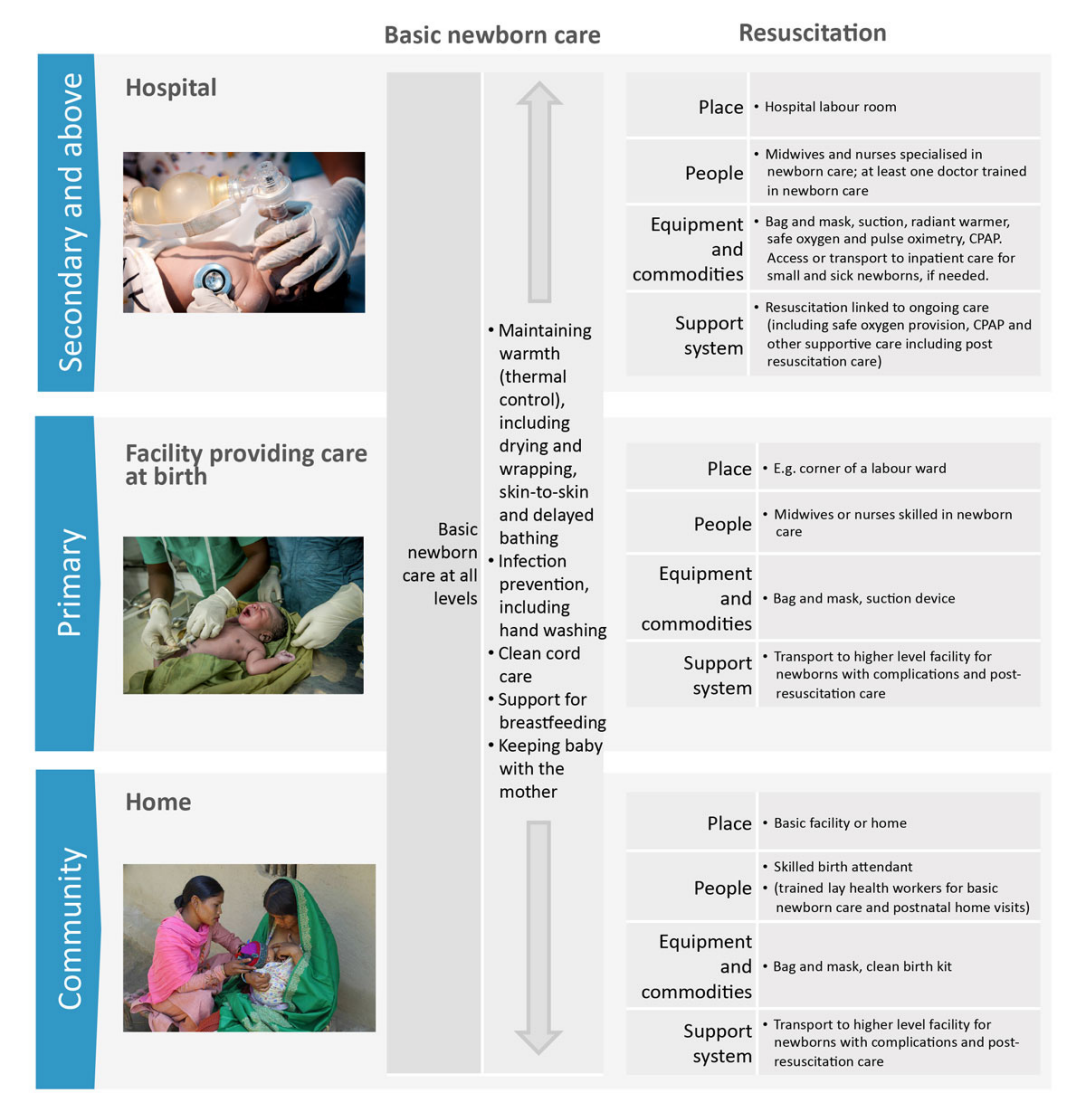
**Basic newborn care and basic neonatal resuscitation, showing health system requirements by level of care**. Hospital level image source: Christena Dowsett/Save the Children. Primary facility image source: Karen Kasmauski/MCSP. Home birth/community level image source: Michael Bisceglie/Save the Children.

BNC processes are simple, essential and implementable by everyone in every setting where births occur (Figure 2). In contrast, resuscitation involves relatively more complex processes and the use of bag and mask equipment is generally restricted to skilled or trained birth attendants [21]. Facility-based clinical interventions provided by educated, trained, licensed, and regulated midwifery personnel (midwives, nurses or doctors) confers the best outcomes for mothers and newborns [20,22-24]. However, in low-resource countries, only an estimated 50% of births take place in health facilities, and these interventions and services have the greatest gaps in coverage, quality and equity in communities that need them most [20,25,26]. Consequently, strategies that strengthen the health system to improve access may increase community utilisation of facility care and improve newborn survival. The substantial impact of improved basic services around the time of birth for women and children has been estimated by the United Nations Commission on Life-Saving Commodities and Lancet Newborn series 2014 [7,27].

Increasing coverage and improving quality of neonatal interventions, especially BNC and resuscitation, are crucial for achieving the goals of the *Every Newborn Action Plan *(ENAP) [4]. Until recently, many low- and middle- income countries (LMIC) lacked appropriate policies and guidelines for providing quality health care to newborns [28-30]. Renewed commitments by governments and partners to the UN Secretary-General's Global Strategy for Women's and Children's Health and to Every Woman, Every Child initiative have led to development of policies and strategies to guide implementation of effective interventions at scale. Also, there is increasing consensus on the content of basic newborn care and resuscitation at and around the time of birth at all levels of health service delivery [8,22,31]. We now need concerted effort to address health system bottlenecks to achieving the goals of ENAP including accelerating universal coverage of quality BNC to all newborns and effective resuscitation to newborns who are unable to initiate breathing at birth.

The objectives of the paper are to:

1. Use a 12-country analysis to explore health system bottlenecks affecting the scale up of basic newborn care and resuscitation

2. Present strategies to overcome the most significant bottlenecks including learning from the 12-country analyses, literature review and programme experience

3. Discuss policy and programmatic implications and propose priority actions for programme scale up.

## Methods

The study used quantitative and qualitative research methods to collect information, assess health system bottlenecks and identify solutions to scale up of maternal and newborn care interventions in 12 countries: Afghanistan, Cameroon, Democratic Republic of Congo (DRC), Kenya, Malawi, Nigeria, Uganda, Bangladesh, India, Nepal, Pakistan and Vietnam.

### Data collection

The maternal-newborn bottleneck analysis tool was developed to assist countries in the identification of bottlenecks to the provision and scale up of maternal and newborn health interventions across the seven health system building blocks as described previously [22,30]. The tool was utilised during a series of national consultations supported by the global Every Newborn Steering Group between July 1^st ^and December 31^st, ^2013 (Additional file 1). The workshops for each country included participants from national ministries of health, UN agencies, the private sector, non-governmental organisations (NGOs), professional bodies, academia, bilateral agencies and other stakeholders. For each workshop, a facilitator oriented on the tool coordinated the process and guided groups to reach consensus on the specific bottlenecks for each health system building block. This paper, fourth in the series, focuses on the provision of basic newborn care and resuscitation both delivered at and around the time of birth.

For the purpose of this bottleneck analysis, tracer interventions were selected for their impact on neonatal outcomes. BNC tracer interventions were cleanliness and cord care, thermal care and support for breastfeeding (Additional file 1). Cleanliness includes sterile birth procedures and hygienic newborn care practices, specifically cord care [10]. Thermal care includes maintaining a warm chain at and around the time of birth to prevent hypothermia, an invisible cause of morbidity and mortality, especially in premature and low birth weight babies [12,32]. Early initiation and exclusivity of breastfeeding is protective to the mother and newborn [33,34]. Establishing effective resuscitation for newborns who need assistance to breathe at birth requires competent work force and commodities; therefore, the tracer intervention selected for basic resuscitation was ventilation by bag and mask (Additional file 1). Effective resuscitation saves lives and reduces long-term disability [17,35]. Other essential preventive interventions (Figure 1) were not the focus of this analysis.

### Data analysis methods

Bottlenecks for each health system building block were graded using one of the following options: not a bottleneck (=1), minor bottleneck (=2), significant bottleneck (=3), or **very major **bottleneck (=4). Data received from each country were analysed and the graded health system building blocks were converted into heat maps. Countries that categorised health system bottlenecks as significant or very major were grouped by mortality contexts (Neonatal Mortality Rate (NMR) <30 deaths per 1000 live births and NMR ≥30 deaths per 1000 live births) and region (countries in Africa and countries in Asia).

The context specific solutions from the countries were categorised into thematic areas linked to specific bottlenecks (Tables S1, S2, S3 and S4, additional file 2; Table 1). We undertook a literature review to identify further case studies, evidence for proposed solutions, and other evidence-based strategies for each tracer intervention. For more detailed analysis of the steps taken to analyse the intervention-specific bottlenecks, please refer to the overview paper [30].

## Results

**Figure 3:**
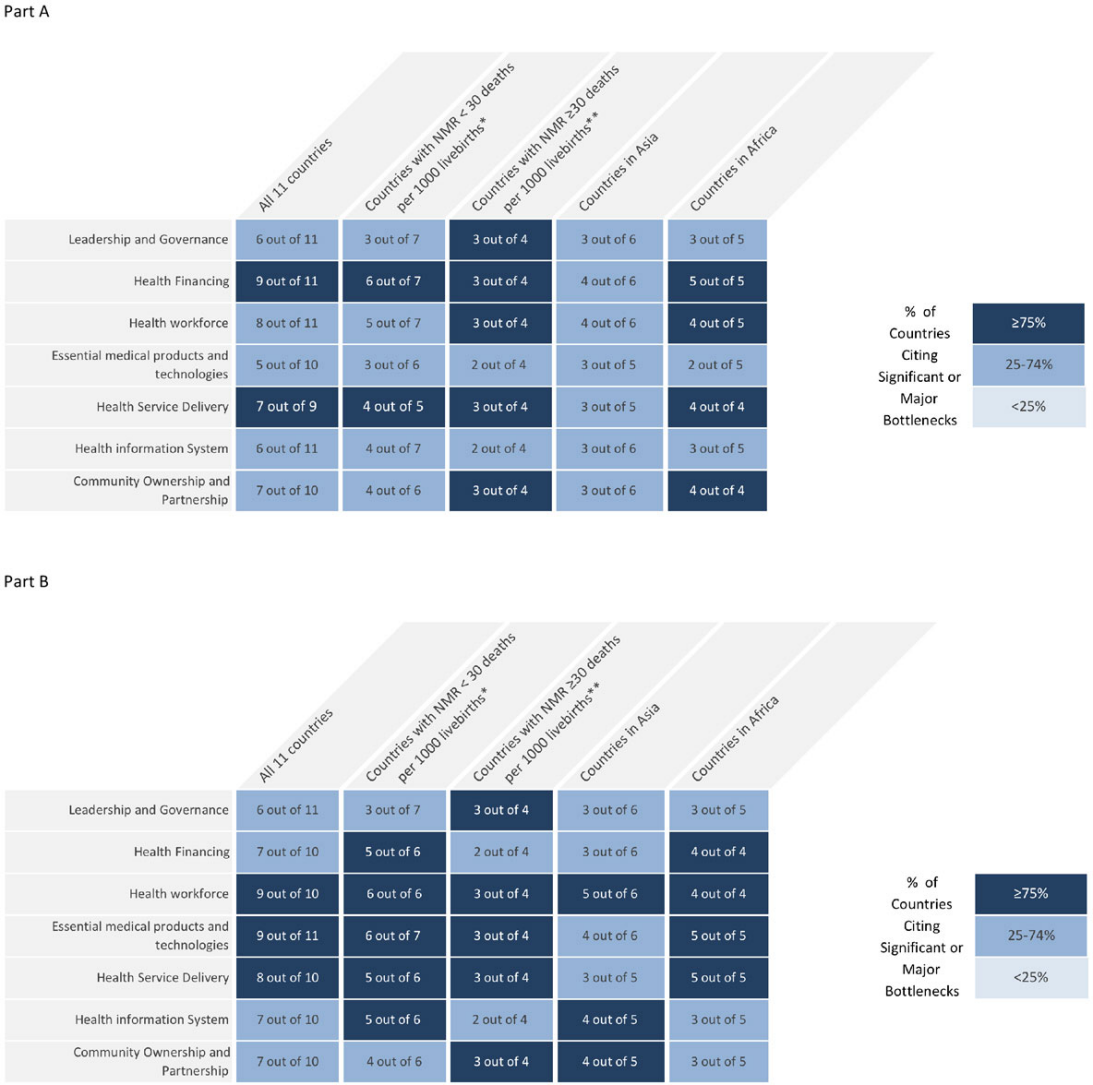
**Very major or significant health system bottlenecks for basic newborn care and neonatal resuscitation**. NMR: Neonatal Mortality Rate. *Cameroon, Kenya, Malawi, Uganda, Bangladesh, Nepal, Vietnam. **Democratic Republic of Congo, Nigeria, Afghanistan, India, Pakistan. See additional file 2 for more details. Part A: Grading according to very major or significant health system bottlenecks for basic newborn care as reported by eleven countries combined. Part B: Grading according to very major or significant health system bottlenecks for neonatal resuscitation as reported by eleven countries combined.

**Figure 4:**
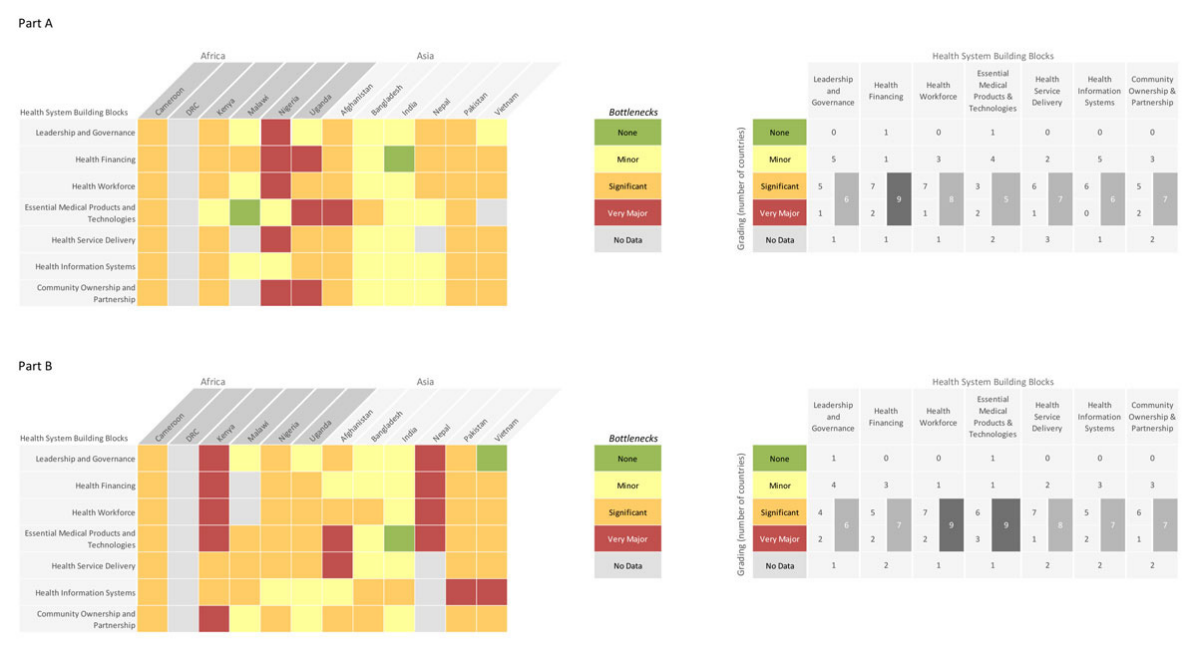
**Individual country grading of health system bottlenecks for basic newborn care and neonatal resuscitation**. DRC: Democratic Republic of the Congo. Part A: Heat map showing individual country grading of health system bottlenecks for basic newborn care and table showing total number of countries grading significant or major bottleneck for calculating priority building blocks. Part B: Heat map showing individual country grading of health system bottlenecks for neonatal resuscitation and table showing total number of countries grading significant or major bottleneck for calculating priority building blocks.

All country workshop teams (Afghanistan, Cameroon, Kenya, Malawi, Nigeria, Uganda, Bangladesh, Nepal, Pakistan, India and Vietnam) except Democratic Republic of Congo (DRC) submitted quantitative data on the grading of health system building blocks (Figures 3 and 4). A country was not included in the quantitative analysis of a health system building block if it did not provide grading data, however, the perceived bottlenecks and solutions were included in the qualitative analysis. Therefore, although workshop participants from DRC did not record any grading of their bottlenecks their qualitative data was included in the analysis along with the other 11 countries (Tables S1, S2, S3 and S4, additional file 2).

Of the eleven country workshop teams that graded the bottlenecks, Afghanistan, Cameroon, Kenya, Malawi, Nigeria, Uganda, Bangladesh, Nepal and Vietnam returned national level responses. Pakistan provided subnational data from all provinces - complete data was received from Gilgit-Baltisan, Azad Jammu and Kashmir, Khayber Pakhtun, Baluchistan, and Punjab but incomplete data was received from Sindh (Figures S1 and S2, additional file 2). India returned subnational data from three states: Andhra Pradesh, Odisha and Rajasthan (Figures S1 and S2, additional file 2). Three country teams (Malawi, Nepal, and Vietnam) provided incomplete data for BNC (Figure 4a), and 2 teams (Malawi and Nepal) provided incomplete data for neonatal resuscitation (Figure 4b).

Overall, bottlenecks for BNC (Figures 3a and 4a) were graded less severely than for neonatal resuscitation (Figures 3b and 4b). Country workshop participants in Africa reported more significant or very major BNC and resuscitation bottlenecks than participants in Asia. The health system building blocks frequently graded as very major or significant were financing, service delivery and workforce for BNC. For neonatal resuscitation the workforce, essential medical products and technologies (also referred to as commodities), and service delivery were most frequently graded as significant or very major. Higher burden country workshop participants (Nigeria, Afghanistan and Pakistan but not India) tended to grade their bottlenecks more severely. Among the 12 countries, the India country team graded the bottlenecks in their health system least severely for both BNC and neonatal resuscitation.

Bag and mask ventilation had the most bottlenecks of the four tracer interventions in this analysis. Country teams provided more solutions for BNC tracer interventions than for neonatal resuscitation. A summary (Table 1) and details (Tables S1, S2, S3 and S4, additional file 2) of BNC and resuscitation health system bottlenecks and proposed solutions are presented.

**Table 1:** Health system bottlenecks to basic newborn care and basic neonatal resuscitation in 12 countries: proposed solutions and evidence.

Health system Building blocks	Bottleneck Category	Number of countries		Proposed solution themes	Evidence for proposed solutions
		**BNC**	**NR**		

Leadership and Governance	Policy: lacking; not updated; poorly disseminated or implemented	6	5	• Update policy and disseminate to district level	Implementation of policies that improve maternal outcomes may improve neonatal outcomes [44]
	Guidelines: unavailable; not updated; poorly disseminated or implemented	8	9	• Integrate facility and community care, improve public-private partnership and implement monitoring mechanisms at all levels/sectors	Improved private-public partnership increases access to institutional perinatal services [43,69]
	Weak enforcement of policy/guidelines on breastfeeding and breast milk substitutes	5	-	• Develop, regularly update and disseminate guidelines and standards	
	Most births occurring at home/attended by unskilled workforce	-	2	• Advocacy to leaders of health facilities on newborn health services	
	Poor public-private partnership and private sector compliance to national standards	2	2		

Health financing	Inadequate funding and budget allocation; inadequate financial guidelines at district level	9	12	• Advocacy to increase budgetary allocation and scope of health insurance coverage	Improving insurance coverage increases utilisation of facility maternity services, evidence on quality of care and health outcomes is inconclusive [78]
	High out-of pocket expenditures for maternal and newborn services	7	3	• Equity in budgetary allocation • Disseminate financial guidelines to districts	Removal of user fees (out-of-pocket) does not significantly impact utilisation of services and may not be sustainable [41,79]
	Funding not specific/prioritised for resuscitation	-	3	• Widen scope of health insurance coverage for newborn services and reduce user fees	
	Low insurance coverage for newborn services	1	1	• Targeted funding for resuscitation equipment and re-training of providers	

Health Workforce	Inadequate knowledge and competency	11	9	• Update and harmonise curricula for training institutions; accreditation of training programs (pre- and in- service)	Competency-based training improves community health workers' effectiveness, positively impact community care-seeking behaviour and neonatal outcomes [80,81]
	Inadequate numbers and poor distribution	9	8	• Competency-based approach for training and learning. Refresher courses for resuscitation	In-service training improves knowledge and performance of facility-based workers but variable effect on health outcomes [45,46,82]
	Poor quality of pre-service and in-service training/ refresher courses	8	8	• National workforce mapping; use data for training and mentoring programs	
	Poor supervision and mentorship	5	6	• Monitoring and supervisory system in line with job description and standards of practice	
	Lack of job description and job aids	4	4	• Equity in distribution; reduce reassignment of staff trained in newborn care	

Essential Medical Products and Technologies	Lack of/inadequate supplies and equipment e.g. essential medicines, warmers, bag and mask equipment	5	9	• Implement policy on essential drugs and commodities especially chlorhexidine	Provision of quality equipment and supplies at point of use improves quality of care [83]
	Inadequate procurement/logistics supply system	4	10	• Logistic and supply management system to improve commodities availability at district level	
	Poor standards/quality of supplied equipment	3	5	• Locally manufacture chlorhexidine, use public-private partnership	
	Chlorhexidine not in national drug lists or implemented at district level	8	-	• Adequate needs assessment and due process for procurement including bidding mechanisms	

Health Service Delivery	Service unavailable; poor coverage/ geographic access	7	6	• Develop and implement referral and transportation mechanisms for newborns	Well-integrated health system improves health outcomes [84]
	Ineffective referral mechanisms; poor linkages between community and health facility/ follow-up services	9	7	• Multi-sectorial collaboration to improve access, sanitation and infrastructure	Supportive supervision and quality perinatal audit and reviews improve adherence to standards and effectiveness of care [47,48]
	Poor quality of care (adherence to standards for hygiene and resuscitation, monitoring mechanisms, health worker attitudes)	7	5	• Continuous quality improvement at district level including supportive supervision and perinatal audit	Accreditation of facilities providing delivery services improves outcome for newborns [65,66]
	Inadequate postnatal care and follow-up / outreach services	8	-	• Accreditation of facilities using a standard process	
	Weak public private partnership/ poor collaboration	4	-		

Health Management Information System (HMIS)	Newborn indicators not captured in national HMIS and reports	9	8	• Update HMIS and integrate clearly defined newborn indicators through consultative national meetings	Standardised indicators improve assessment, decision-making and quality of care [85]
	Inadequate or complicated tools for information system and reporting; limited or poor quality of data	5	4	• Develop monitoring tools, set up surveillance system for important indicators	Effective perinatal audit programs improves health professionals' practices and neonatal outcomes [47,74]
	Poor documentation of clinical practice and implementation of perinatal/clinical audits and reviews	6	9	• Train and retrain HMIS personnel; disseminate protocols on perinatal audit to district level • Use local data at district meetings, for quality improvement and decision-making	

Community Ownership and Partnership	Poor community and male involvement to facilitate care seeking	6	6	• Multiple channels of information dissemination on importance of BNC and resuscitation	Adequate engagement and information to communities reduces major barriers to access and utilisation of facility-based services and improves health outcomes [86]
	Limited community awareness and inadequate strategies to facilitate knowledge about newborn issues	6	6	• Advocacy and engagement of community leaders to sensitise the community	Community mobilisation and training of community health workers including traditional birth attendants reduces perinatal mortality, improves referrals and early initiation of breast feeding [14]
	Socio-cultural and gender barriers / challenges faced by mothers	9	4	• Community representation at facility audit meetings	
	Access constraints (distance, cost of travel and care)	7	4	• Improve community and facility workforce linkages, provide context-appropriate IEC tools	
	Limited knowledge and communication skills of health providers and lack of IEC materials in appropriate local languages	2	5	• Train and retrain community workforce especially on communication skills	

BNC: Basic Newborn Care; NR: Neonatal Resuscitation; IEC: Information Education and Communication

## Leadership and governance bottlenecks and solutions

High neonatal mortality (NMR ≥30) burden settings graded leadership and governance barriers more severely (Figures 3 and 4). Overall, country teams described similar leadership bottlenecks in the qualitative data (Table 1). Developing, disseminating and implementing appropriate policies and guidelines for all levels and sectors of maternal-newborn services were the major perceived governance bottlenecks for countries. Workshop participants in Vietnam reported inadequate awareness among national leaders. Bangladesh workshop participants described the policy permitting only skilled birth attendants to perform resuscitation as a major barrier to scaling up the intervention given that two-thirds of national births are attended by non-skilled workers.

Solutions proposed by country teams in Africa and Asia were largely similar (Table 1). The India team proposed establishment of a national newborn technical group linked to the Ministry of Health, to review and update existing policies and guidelines, harmonise related strategies and set national standards for BNC and resuscitation. The Kenya team proposed a national implementation committee to monitor implementation of national newborn action plans.

## Health financing bottlenecks and solutions

Most country workshop teams perceived financing as a significant or very major barrier to provision of BNC (5 of 5 in Africa, 4 of 6 in Asia) and resuscitation (4 of 4 in Africa, 3 of 6 in Asia). India workshop participants did not perceive health financing as a barrier for BNC services (Figure 4a). The barrier posed by out-of-pocket expenditure was perceived as a greater challenge for BNC (4 of 6 in Africa, 3 of 6 in Asia) than for resuscitation (2 of 6 in Africa, 1 of 6 in Asia) (Table 1, Tables S1 and S2, additional file 2).

Nigeria was the only country that reported low health insurance coverage as barrier to scaling up newborn interventions. Nonetheless, six country teams recommended widening the scope of health insurance or other financial plans for maternal and newborn services including insurance coverage for specialised/intensive care services for newborns identified with complications during the first day assessment and post-resuscitation care (Table S3 and S4, additional file 2). Improvements in funding procedures at district level and targeted funding for regular in-services refresher training and replacement of supplies for neonatal resuscitation were some of the specific solutions proposed.

## Health workforce bottlenecks and solutions

Bottlenecks within the workforce were graded as very major or significant for both BNC (8 countries) and resuscitation (9 countries) (Figure 4). All 12 country teams identified challenges within their workforce in the qualitative data. Specifically, skills, overall numbers, quality of training (pre-service and in-service), and supportive supervision were described as inadequate and available workforce were distributed inequitably (Table 1; Table S1 and S2, additional file 2). These bottlenecks were more critical for neonatal resuscitation services at district level. Lack of job aids and job description were barriers reported by participants in Asia (4 of 6 countries).

Proposed solutions to improve health workforce capacity were largely similar for Africa and Asia and include pre- and in- service training strategies, improved conditions of service for the workforce, and improved human resource management (Table 1). Provision of job descriptions and job aids and implementation of standards of practice were suggested measures to improve workforce productivity and effectiveness of care.

## Essential medical products and technologies bottlenecks and solutions

Country workshop teams graded bottlenecks associated with commodities for resuscitation more severely (5 of 5 in Africa, 4 of 6 in Asia) than for BNC (Figure 4). Qualitative data show that bottlenecks within the procurement process and supply chain were particularly challenging for most countries (Table 1; Table S1 and S2, additional file 2). Specifically, teams described the procurement process as complex and ineffective, the consultation process with end users about specification of commodities to be purchased is not being implemented, and vital equipment for resuscitation including bag and mask equipment and basic supplies are not readily available at the point of use. Lack of chlorhexidine at the point of use was also perceived as a barrier to providing quality BNC (Table 1).

Although all 12 country teams reported bottlenecks with commodities for neonatal resuscitation in the qualitative analysis, only 5 country teams offered solutions to these bottlenecks (Tables S3 and S4, additional file 2). Proposed solutions include personnel training, especially program managers and procurement officers; improving supply management strategies; and policy implementation on essential equipment and drugs for newborn care particularly chlorhexidine digluconate. The teams recommended that procurement of commodities should be based on needs assessment and consultations with end users to ensure that appropriate products are provided (Table 1).

## Health service delivery bottlenecks and solutions

All country teams that provided quantitative data except India and Bangladesh graded service delivery bottlenecks for both BNC and resuscitation as significant or very major (Figure 4). Poor linkages between health facilities and between the community and health facility, including referral-feedback systems, transportation, follow-up, and outreach programs were reported as barriers to effective health service delivery (Table 1; Table S1 and S2, additional file 2). There is limited coverage of neonatal resuscitation at district level in Cameroon and Vietnam because of inadequate workforce and infrastructure including equipment for providing the service. Most country teams described the quality of care in health facilities where delivery and newborn services are available as poor or inadequate due to low hygienic standards, poor health worker attitude, poor adherence to guidelines and protocols, and non-implementation of quality improvement activities.

Country teams proposed a holistic approach to overcoming these barriers including, extending government training programs to the private sector, health facility accreditation, strengthening capacity of health facilities through supportive supervision and monitoring, promoting in utero referral of fetuses at risk to higher level facilities, and effective systems for continuous quality improvement including clinical reviews and perinatal audit (Table 1; Table S3 and S4, additional file 2).

## Health information system bottlenecks and solutions

The absence of newborn indicators in the national HMIS was reported by most country workshop teams (Table 1). National HMIS do not capture basic newborn care and resuscitation activities and the cause of newborn deaths. Documentation of newborn clinical services especially neonatal resuscitation is generally poor, clinical reviews and perinatal audit are poorly implemented and available data are inadequate for action and decision-making. Country workshop teams in Pakistan and Vietnam graded HMIS severely for neonatal resuscitation (Figure 4b). In Nepal and Vietnam, teams reported that the available data tools were overly complex for data management personnel at district level to effectively integrate newborn care activities into the HMIS. The solutions provided by other country teams for improving data quality suggest that the barriers highlighted by Nepal and Vietnam were also significant for other countries in this analysis.

Workshop participants emphasised the need for newborn indicators that can feasibly measure and accurately capture BNC and resuscitation activities at all levels and sectors that provide maternal and newborn health services. They suggested training platforms for national and district level HMIS personnel and cell phone-based mHealth systems to track home deliveries and link community health workers to health facilities (Table 1; Table S3 and S4, additional file 2).

## Community ownership and partnership bottlenecks and solutions

All workshop teams in Africa (4 out of 4) and fewer in Asia (3 out of 6) graded community ownership and partnership as having significant or very major bottlenecks for BNC (Figure 4a). Inadequate involvement of communities with the health system and context-specific socio-cultural factors were the most common causes of perceived bottlenecks (Table 1; Table S1 and S2, additional file 2).

Proposed solutions include advocacy and communication strategies to reach various categories of leaders and community groups, and national and district level sensitisation and educational programs to improve population knowledge on perinatal services especially on neonatal emergencies (Table 1).

## Discussion

To our knowledge, this article is the first systematic analysis of bottlenecks and solutions to BNC and resuscitation around the time of birth from 12 low and middle income countries which together account for around half of global maternal and newborn deaths. Consistent with previous analysis, we show that out of 9 maternal and newborn intervention packages, BNC has the least perceived bottlenecks to scale up [22,30]. Nonetheless, inadequate financing, service delivery and workforce performance are important fundamental challenges hindering scale up of even very basic newborn care for most countries. The analysis underscores the common challenges countries encounter with BNC and resuscitation services and the importance of linking facility and community services.

The advantages and limitations of the methodology in this analysis have been addressed elsewhere [30]. Although resuscitation was graded more severely, the bottlenecks described for BNC and resuscitation were largely similar. Fewer solutions were proposed for resuscitation, but solutions proposed for BNC were rationally linked to resuscitation at the time of birth. The challenges with neonatal resuscitation services in low-resource settings are likely best appreciated by clinical practitioners; inadequate representation of such practitioners in some of the country workshops may account for the few solutions provided for resuscitation.

Consistent with previous studies, we show that resource constraints, including workforce, financing and commodities are barriers to optimal care in LMIC [22,36]. We also confirm that gaps in the process of care hinder delivery of quality services [37,38]. For example, poor workforce competency limits the application of technologies such as bag and mask ventilation and use of HMIS technologies at district level.

### Health financing priority actions

Financing challenges and solutions identified by workshop teams were not specific for BNC and resuscitation, but rather part of the larger national infrastructural and development funding deficits that also affects health care services [30]. There is limited budget for renovating infrastructure for maternal and newborn services, in-service training, regular provision of equipment and supplies, and incentives for health workers. Efficient use of the proposed targeted funding for basic supplies and training the workforce, especially on neonatal resuscitation, may improve retention of clinical skills and health outcomes [39,40].

High out-of-pocket expenditure was perceived as a barrier for BNC by more country teams (7 countries) than for resuscitation (3 countries) even though neonatal resuscitation requires more process inputs (Figure 2). This rather unexpected finding could be explained by aggregate costing of birth/delivery services. User fees (out-of-pocket) at point of care may not significantly reduce access to facility services, however, countries considering removal of user fees should provide alternative sustainable sources of funding for human resource and infrastructural needs that depend on user fees [41]. Advocacy and funding schemes to improve facility-based newborn services should be extended to specialised/intensive care services for post-resuscitation care and other newborn complications [42].

### Health workforce priority actions

The performance of the workforce is the critical constraint in all health system building blocks for all 12 countries. Countries lack sufficient numbers of competent workers especially midwives and nurses to deliver quality care to newborns, particularly basic neonatal resuscitation at lower levels of health service delivery.

#### Health workforce performance

Advocacy for leaders of health facilities on the needs of newborns was proposed as a solution for overcoming health system barriers. This implies that improving leadership skills of facility administrators with a focus on the needs of mothers and newborns is an opportunity to overcome perceived barriers. Leadership training and higher level support with tools to institute organisational changes that create an environment for quality maternal and newborn services in public- and private- sector health facilities is essential, especially for high burden countries [23,43].

The health workforce of countries in this analysis can institute change and meet the needs of mothers and newborns around the time of birth [44,45]. Skills training, guidelines, support, opportunities for collective action and process improvement, commodities, motivation, and job descriptions are needed for the existing workforce to improve performance and impact health outcomes [43,46-48]. Redistribution of skilled birth attendants, especially midwives, to rural settings with adequate incentives, and shifting tasks to appropriate lower level workers with defined roles and adequate supervision may reduce inequities [21]. Inadequate numbers of skilled providers can be addressed by training more midwives and innovative context-based strategies to ensure retention of skilled workforce in challenging settings [23,49]. Also, countries should consider rectifying shortages in higher level workforce (including specialist midwives, neonatal nurses, obstetricians, paediatricians and neonatologists) as this core group is essential for building capacity of a competent workforce for the medium and long term.

An estimated 50 million births (40% of the total number of births worldwide) occur outside health facilities [6]. In this analysis, Nigeria and Bangladesh report that most births neither occur in health facilities nor are attended by skilled birth attendants. In Asia, an estimated two thirds of home births are attended by a traditional birth attendant (TBA), however, in Africa, most births that occur outside facilities are at home alone (without any attendance) [6]. Given the potential to reduce inequities by extending care to underserved populations, the use of trained lay health workers, including TBAs, is often proposed as a solution. As circumstances vary by region, mortality context, local culture and existing health infrastructure, such an approach requires careful consideration of who is currently attending the births and how they are linked to the health system. Emphasis should primarily be placed on training in basic newborn care practices [17]. Whilst some studies have shown that training of lay health workers, including TBAs, on neonatal resuscitation can be done successfully [21,47], there are cost, effectiveness and sustainability issues, especially where the attendants may not see sufficient volume of births to be able to maintain skills. Careful evaluation is needed and should be based on the specific implementation context. Given the synergies between maternal and newborn health, however, investments in health system strengthening should give priority to skilled care at birth from which both mother and child will benefit.

#### Competency-based training

Competency-based approach to pre-service and in-service education is critical for sustainable, wider scale implementation of BNC and resuscitation [46,50]. Training programs should ideally incorporate monitoring and evaluation tools for assessing program cost-effectiveness and impact on health outcomes [51,52]. The curriculum should address context-specific barriers and technical/procedural skills e.g. ability to perform neonatal resuscitation. It should also ensure that higher order skills including leadership, communication, clinical decision-making, data management, quality improvement, and ethical practices are addressed.

Many studies proclaim the success of stand-alone training programs, but these successes tend to be short-lived because of poor policy and leadership support and inappropriate educational approaches. Programme experience from The Western Pacific Region show that a systematic approach that incorporates in-depth quality situation analysis, wide consultation and inclusiveness of key stakeholders in updating clinical protocols and planning training programs have potential to create sustainable change [50]. Lessons can be learnt from approaches that incorporate evidence-based science and innovative educational methods to build competency and confidence in the workforce [53].

The national technical or implementation group proposed by India and Kenya could work with ministries of health and education to update and harmonise the curricula of training institutions, set standards for accreditation, provide guidance on innovative training approaches that address local gaps, and monitor implementation of national plans in training institutions and health facilities. These measures would ensure consistency in standards of care in the public and private sector not only for BNC and basic neonatal resuscitation but also for small and sick newborns [42].

### Essential medical products and technologies priority actions

Commodities required for BNC and resuscitation should be available everywhere births occur; however, many LMIC health facilities lack these commodities and the prerequisites for hygienic practices such as clean water, soap, gloves and alcohol rub. Effective logistic information systems that monitor local and national uptake of commodities and transparent procurement procedures could address some of the perceived barriers [54].

#### Cleanliness and cord care

Perceived barriers to delivering quality care in this analysis include inadequate supplies for general cleanliness and clean birth procedures, and non-adherence to standards for cleanliness and procedures for use of available commodities by health workers [55]. Unhygienic practices in the use of technologies (including radiant warmers, bag and mask equipment, weighing scales and thermometers), and non-adherence to basic rules of hygiene such as removing jewellery and not using cell phones during patient care, could be addressed by training, supportive supervision and enforcement of infection control rules to change health providers' attitude [56,57].

**Figure 5:**
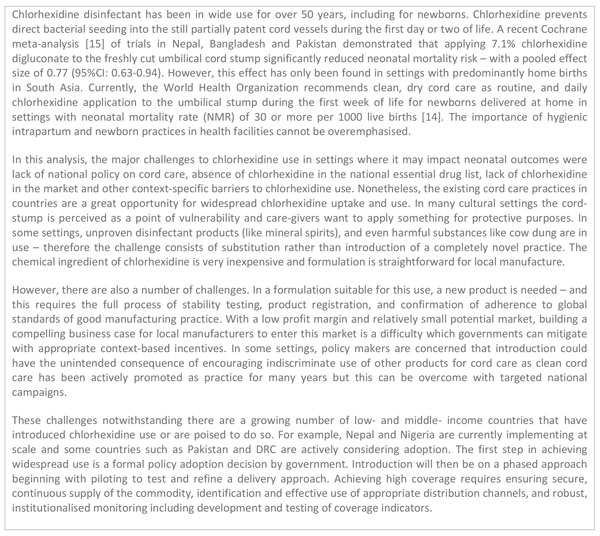
**Implementing chlorhexidine use for cord care**. NMR: Neonatal Mortality Rate. DRC: Democratic Republic of the Congo.

Clean delivery practices can be improved by effective supervision and provision of essential commodities for hand hygiene e.g. water and soap, and clean delivery kits [55]; but, behaviour change is key to sustainable clean delivery practices. Although evidence on the impact of clean delivery kits on neonatal mortality is inconsistent [58], analysis of data from Bangladesh, Nepal and India show that use of each additional content of the kit e.g. sterilised blade to cut the cord, was associated with reduction in neonatal mortality [59]. Chlorhexidine digluconate use for cord care effectively reduces infection-associated deaths in settings where most births are attended by unskilled workforce [15]. We suggest strategies to overcome challenges to scale-up use of chlorhexidine digluconate (Figure 5).

#### Essential commodities and provision of basic neonatal resuscitation services

Every country that participated in this analysis had challenges with providing resuscitation services at scale. These challenges were mostly related to workforce competency, availability and use of technologies, and health service delivery; but the availability and use of technology/commodities was the pivotal barrier. Birth attendants lack the requisite skills to provide effective intrapartum fetal monitoring and newborn resuscitation [55]. Context-specific schemes that address health worker beliefs and practices, facility policies that support incorrect practices, and other health system bottlenecks especially concerning the availability of bag and mask at point of use should be explored.

**Figure 6:**
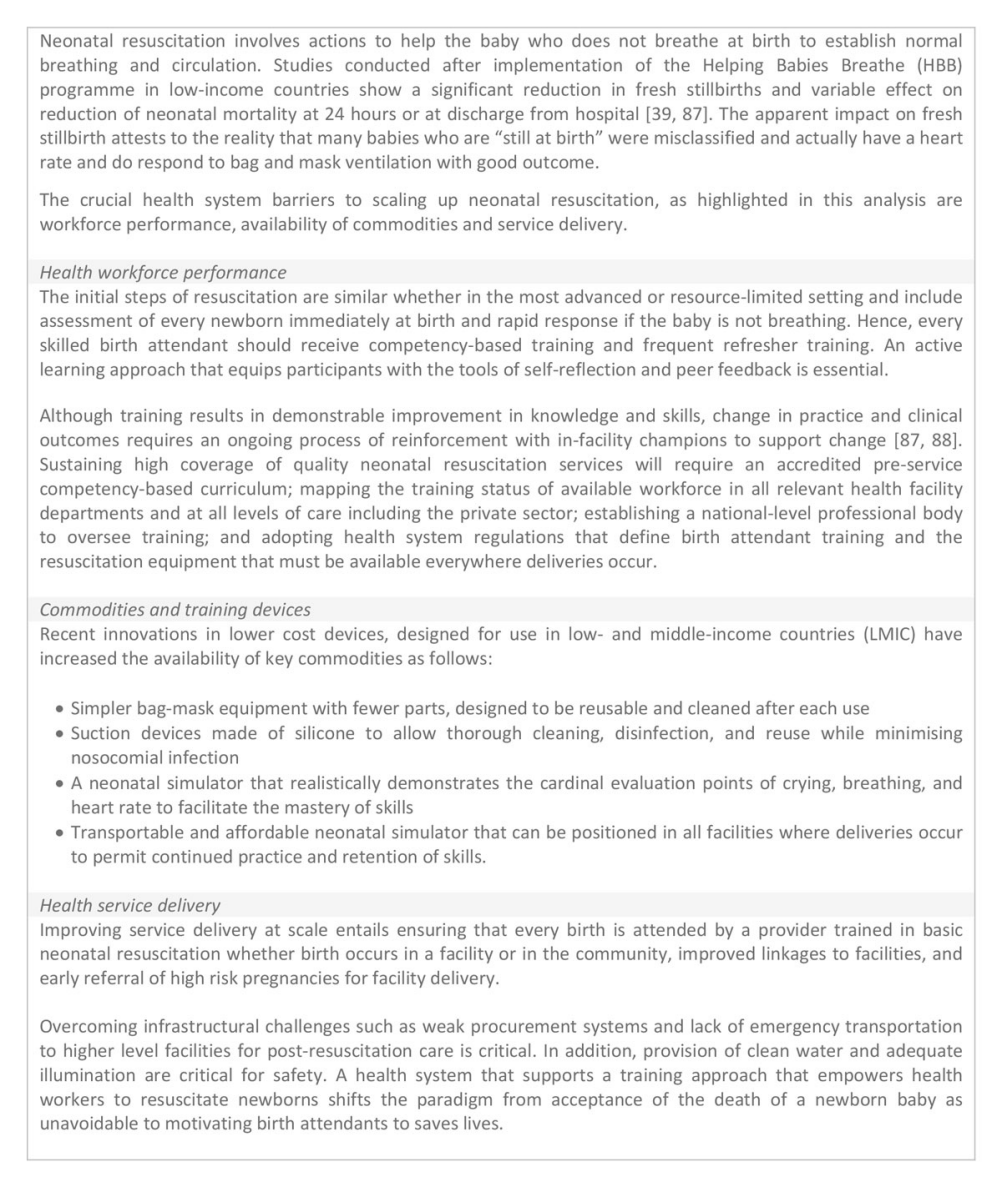
**Scaling up universal access to neonatal resuscitation**. HBB: helping babies breathe. LMIC: low and middle income countries

Birth attendants usually show significant improvement in knowledge after training on neonatal resuscitation but acquisition and retention of skills is still a major challenge globally [38,60,61]. Sustaining competency in rural settings with lower volume facilities, few deliveries, less exposure and practice poses greater challenge. We present an approach to address workforce competency and availability and use of basic commodities for newborn resuscitation (Figure 6). Global initiatives should not only focus on providing bag and mask equipment and other supplies for resuscitation; these initiatives should also address health service delivery challenges. Specifically, newborns requiring extra care after resuscitation need timely referral and transportation to specialised care centres where appropriate inpatient care can be provided; resources should be provided to make this happen [42].

### Health service delivery priority actions

**Figure 7:**
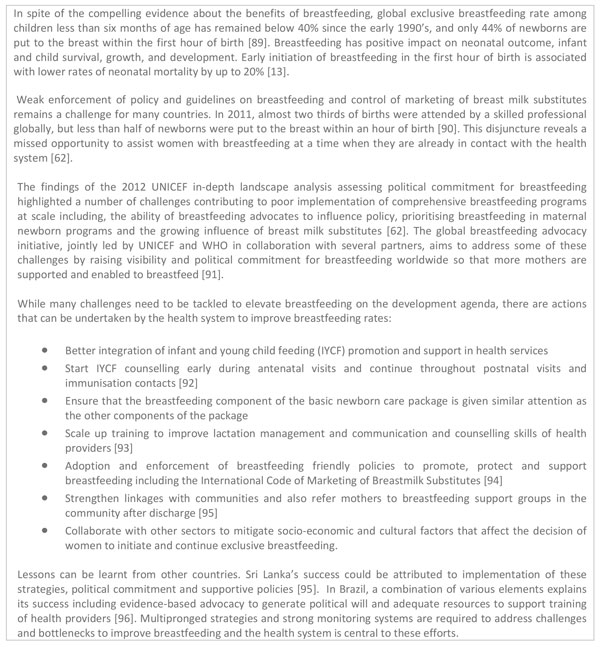
**Early and exclusive breastfeeding for every newborn**. IYCF: infant and young child feeding. UNICEF: United Nations International Children's Emergency Fund. WHO: World Health Organization

Implementing evidence-based and context-appropriate policies and guidelines at all levels of care and the private sector is the important leadership and governance challenge reported by country teams, especially those with high neonatal mortality burden. Specifically, policy on breastfeeding practices is not consistently implemented despite the evidence for benefits of early initiation and exclusivity of breastfeeding [14,62]. We suggest strategies to improve breastfeeding practices (Figure 7).

Coverage targets for facility births in LMIC are gradually being achieved but the quality of care has been described as substandard. Implementing appropriate accountability mechanisms, job aids, and effective clinical audits and reviews may overcome some of the lapses identified in this analysis is recommended [63]. Skilled birth attendants are few and overworked but adherence to standards may not increase workload if care is well organised [64]. Creating centres of excellence and formulating standards for external evaluation and accreditation could improve the quality of health service delivery [65,66].

Also, improving the organisation of antenatal-perinatal care and referral systems to enhance in utero referral of fetuses at risk to higher level facilities will improve health outcomes for mothers and newborns [67,68]. Furthermore, improving public-private sector collaboration, engaging other sectors of government that influence access and quality of health care services, and community involvement especially in hard-to-reach areas, will improve access and minimise disparities in health service delivery [43,69,70].

### Other priority actions

Leadership and governance: National governments' endorsement of the goals of *Committing to Child Survival: A Promise Renewed *calls for concerted governmental involvement in the engagement and coordination of all stakeholders including development partners and communities to ensure quality care for mothers and children. Political, religious and traditional leaders can significantly influence community attitudes toward facility-based perinatal services and quality of care in health facilities if global and country-based evidence for policy is effectively communicated to them [71,72], e.g. the long-term economic and health effects of promoting breastfeeding and reducing intrapartum-related disabilities. The role of leaders in all health system building blocks cannot be overemphasised.

Health management information systems: Lack of appropriate newborn indicators for monitoring BNC and resuscitation and inadequate capacity of HMIS personnel to use available tools hinder adequate evaluation of newborn services, appropriate planning, and decision-making [73]. These findings underscore the value of training and retraining HMIS personnel and emphasise the importance of evaluating the process of care with all cadres of the workforce through audit and feedback mechanisms [63,74].

Community ownership and participation: Available evidence supports the multi-pronged approach proposed by workshop participants for engaging communities and includes: community mobilisation strategies; equipping community health workers with adequate skills and tools to effectively communicate the health needs of mothers and newborns; and improving linkages between health facilities and communities [21,44,75].

### Limitations

The data generated from country workshops came from the consensus views of workshop participants and are, therefore, subjective. Bottlenecks were reported as perceived bottlenecks relative to other health system building blocks. It is likely that some of the country participants may have been involved in the same training programs or supported by same non-governmental organisations, therefore, some responses may reflect "group thinking". The quality and amount of information extracted from the workshops varied depending on the capacity of the facilitator, and the knowledge and expertise of participants on health system issues and newborn care. Consequently, there may be instances where known health system challenges or deficits based on robust quantitative national data may be in conflict with the perceived bottleneck grading in this analysis. For example, weak referral systems and poor quality commodities were bottlenecks mostly identified by Asian country teams (Table S1 and S2, additional file 2). These bottlenecks occur in Africa, but African participants may have placed less subjective value on these factors than more severe bottlenecks in their health system. Also, workshop participants provided minimal data (lack of radiant warmers) on thermal care tracer intervention. While this limitation may be attributed to the tool and expertise of workshop participants, it underscores the invisibility of hypothermia as a cause of neonatal morbidity and mortality.

### Future agenda

ENAP offers governments and stakeholders a roadmap to rally behind a global plan to reduce neonatal mortality. The global research community has a major role to play. Innovative platforms such as mobile phones and video-guided web-based educational programs are being used to disseminate health messages and reinforce training even in the most remote regions [76]. The cost-effectiveness, sustainability and impact on health outcomes of these approaches need further consideration.

Of the 4 tracer interventions in this analysis, bottlenecks to thermal care were barely reported; implying the invisibility of the harmful effects of hypothermia. Providing a warm chain at birth by drying babies with warm linen in settings without grid power is a challenge. Technological innovation of affordable linen-materials that can provide warmth on contact during the process of drying, resuscitation, and covering the back of low birth weight babies during skin-to-skin contact with their mothers merit study.

Innovative designs in bag and mask equipment to reduce mask leak and provide real-life feedback on the effectiveness of ventilation offer potential to improve quality of training and clinical care. Also, affordable, portable and non-grid dependent technologies for monitoring newborns during resuscitation may improve the effectiveness of the intervention [77]. Advocacy to manufacturers and international organisations for standardisation to ensure that these commodities meet WHO standards is recommended.

Existing evidence linking quality improvement strategies to improved outcomes is limited. Future research would benefit from the inclusion of more appropriate BNC and resuscitation indicators and additional focus on non-facility determinants of health service quality such as community acceptability and equity of care.

## Conclusions

**Figure 8:**
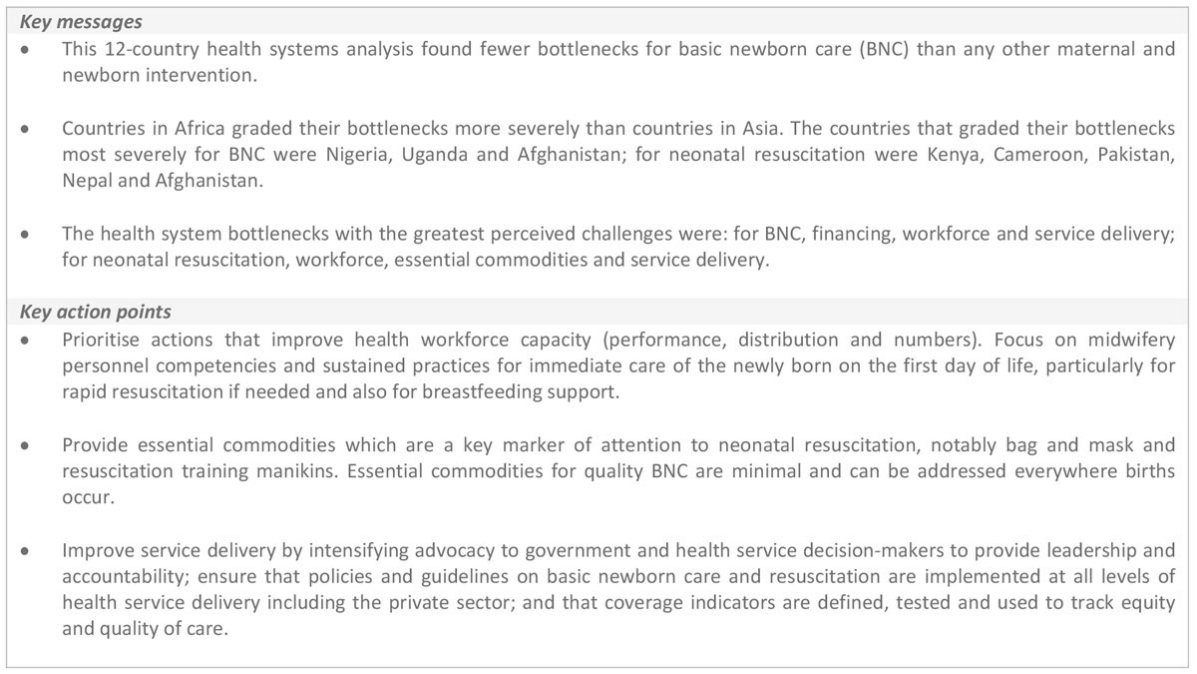
**Key messages and action points for basic newborn care and neonatal resuscitation**. BNC: basic newborn care

Key inter-sectorial players in the provision of newborn care services in 12 countries with high neonatal mortality burden have provided profound insight into difficult to measure factors that hinder progress in the quality and coverage of BNC and resuscitation interventions. The major barriers identified in this analysis can be surmounted and the proposed solutions are implementable if all stakeholders commit to actions that ensure quality survival of newborns especially on the first day of life. Policies and guidelines need to be implemented effectively to ensure that standards are maintained at all levels of maternal and newborn services including the private sector. This will require effective leadership, targeted funding, improved human resource performance and placement, and well-organised client-centred health services that effectively mobilise communities to participate in national strategies to improve health outcome for mothers and newborns. The information provided in this paper should help to create awareness for countries to evaluate their health systems and develop strategies to reduce barriers to effective coverage of BNC and resuscitation (Figure 8).

## List of abbreviations

BNC: Basic Newborn Care; DRC: Democratic Republic of Congo; ENAP: Every Newborn Action Plan; HMIS: Health Management Information Systems; IEC: Information, Education and Communication; LMIC: Low and Middle Income Countries; NGOs: Non-Governmental Organisations; NMR: Neonatal Mortality Rate; NR: Neonatal Resuscitation; UNICEF: United Nations International Children's Emergency Fund; WHO: World Health Organization.

## Competing interests

The authors have not declared competing interests. The assessment of bottlenecks expressed during consultations reflects the perception of the technical experts and may not be national policy. The authors alone are responsible for the views expressed in this article and they do not necessarily represent the decisions, policy or views of the organisations listed, including WHO.

## Authors' contributions

KED and AS-K were responsible for the overall coordination of the country consultation process, bottleneck analysis tool development, data analysis and reviews of the paper drafts. CE-L was responsible for the data analysis and writing process. JEL and SRX contributed to the tool development and oversaw the writing and reviews of the paper drafts. SGM coordinated the writing and reviews. CN contributed to the data analysis. ACCL, FB, HLS, and SN contributed sections of text. All named authors contributed to paper drafts and approved the final manuscript.

## Supplementary Material

Additional file 1 Bottleneck tool questionnaire. Click here for file

Additional file 2 Supplementary tables, figures and literature search strategy. Click here for file

## Declarations

Publication costs for this supplement was funded by the Bill and Melinda Gates Foundation through a grant to US Fund for UNICEF (Grant ID: OPP1094117), and support from Save the Children's Saving Newborn Lives Programme. Additional funding for the bottleneck analysis was received from USAID (Grant ID: GHA-G-00-07-00007) through UNICEF.

This article has been published as part of *BMC Pregnancy and Childbirth *Volume 15 Supplement 2, 2015: Every Woman, Every Newborn. The full contents of the supplement are available online at http://www.biomedcentral.com/bmcpregnancychildbirth/supplements/15/S2.
