# Supplementary material for: Basic newborn care and neonatal resuscitation: a multi-country analysis of health system bottlenecks and potential solutions
Source: BMC Pregnancy Childbirth. 2015 Sep 11;15(Suppl 2):S4. doi: 10.1186/1471-2393-15-S2-S4 (PMC4577863; doi:10.1186/1471-2393-15-S2-S4)
Supplement: Additional file 2 — Supplementary tables, figures and literature search strategy. [file 1471-2393-15-S2-S4-S2.docx]

Basic newborn care and neonatal resuscitation: a multi-country analysis of health system bottlenecks and solutions

Additional file 2

A. Table S1: Bottlenecks for basic newborn care 2

B. Table S2: Bottlenecks for neonatal resuscitation 5

C. Table S3: Solutions for basic newborn care 9

D. Table S4: Solutions for neonatal resuscitation 27

E. Figure S1: Subnational grading of bottlenecks for basic newborn care 38

F. Figure S2: Subnational grading of bottlenecks for neonatal resuscitation 39

G. Literature search strategy 40

# A. Table S1: Bottlenecks for basic newborn care

| **Health System Building blocks** | **Bottleneck Category** | **Africa** | | | | | | **Asia** | | | | | |
| --- | --- | --- | --- | --- | --- | --- | --- | --- | --- | --- | --- | --- | --- |
|  |  | Cameroon | DRC | Kenya | Malawi | Nigeria | Uganda | Afghanistan | Bangladesh | India | Nepal | Pakistan | Vietnam |
| **Leadership and Governance** | Guidelines unavailable /not updated/ poorly disseminated or implemented |  | ✓ | ✓ | ✓ | ✓ | ✓ | ✓ |  | ✓ |  | ✓ |  |
|  | Poor awareness of leaders /poor infrastructure for newborn care |  |  |  |  |  |  |  |  |  |  |  | ✓ |
|  | Policy lacking /not disseminated or implementation e.g. national policy on chlorhexidine | ✓ |  |  |  | ✓ | ✓ | ✓ |  |  | ✓ | ✓ |  |
|  | Weak enforcement of policy /guidelines on breastfeeding and breast milk substitutes |  |  | ✓ |  |  |  | ✓ |  | ✓ | ✓ | ✓ |  |
|  | Poor public-private partnership /private sector compliance to national standards |  |  |  |  |  |  |  | ✓ | ✓ |  |  |  |
| **Health Financing** | Lack of or inadequate funding/budget allocation and financial guidelines | ✓ | ✓ | ✓ | ✓ |  | ✓ |  | ✓ | ✓ |  | ✓ | ✓ |
|  | Low insurance coverage for newborn services |  |  |  |  | ✓ |  |  |  |  |  |  |  |
|  | High out-of pocket expenditures for maternal and newborn services | ✓ |  |  | ✓ | ✓ |  | ✓ | ✓ |  | ✓ | ✓ |  |
| **Health Workforce** | Poor competency, skills and knowledge on newborn care |  | ✓ | ✓ | ✓ | ✓ | ✓ | ✓ | ✓ | ✓ | ✓ | ✓ | ✓ |
|  | Inadequate number and poor distribution of health workers |  |  | ✓ |  | ✓ | ✓ | ✓ | ✓ | ✓ | ✓ | ✓ | ✓ |
|  | Inadequate in-service and pre-service training/ refresher courses |  | ✓ |  | ✓ | ✓ | ✓ |  | ✓ |  | ✓ | ✓ | ✓ |
|  | Poor supervision and mentorship/ monitoring |  | ✓ | ✓ |  |  |  |  |  | ✓ |  | ✓ | ✓ |
|  | Community health workers not involved/ trained on newborn care | ✓ |  |  |  |  |  | ✓ |  |  |  |  | ✓ |
|  | Poor remuneration/ attitude of workers |  |  | ✓ |  |  |  |  |  | ✓ |  | ✓ |  |
|  | Lack of job description and job aids |  |  |  |  |  |  |  | ✓ |  | ✓ | ✓ | ✓ |
| **Essential Medical Products and Technologies** | Lack of/ inadequate supplies and equipment e.g. essential medicines and warmers |  | ✓ |  |  | ✓ | ✓ |  |  |  |  | ✓ | ✓ |
|  | Chlorhexidine not in national drug lists | ✓ | ✓ | ✓ | ✓ |  |  | ✓ | ✓ |  |  | ✓ | ✓ |
|  | Poor standards / quality of supplied equipment |  |  |  |  |  |  |  |  | ✓ |  | ✓ | ✓ |
|  | Weak procurement and supply chain |  |  |  |  | ✓ |  |  |  | ✓ | ✓ | ✓ |  |
| **Health Service Delivery** | Poor coverage/ geographic access to BNC services | ✓ |  | ✓ | ✓ | ✓ |  | ✓ |  | ✓ |  | ✓ |  |
|  | Ineffective/ weak referral mechanisms and linkages between communities and health facilities |  |  | ✓ |  |  |  | ✓ |  | ✓ | ✓ | ✓ | ✓ |
|  | Weak public private partnership and poor private sector collaboration |  |  | ✓ |  |  |  | ✓ |  | ✓ |  | ✓ |  |
|  | Inadequate postnatal care and follow-up / outreach services | ✓ |  | ✓ | ✓ |  | ✓ | ✓ |  | ✓ | ✓ | ✓ |  |
|  | Poor quality of care (adherence to resuscitation/hygiene standards; monitoring mechanisms; quality assurance systems) | ✓ |  |  |  | ✓ |  | ✓ | ✓ |  | ✓ | ✓ | ✓ |
| **Health Information System** | Missing or complicated tools for information system and reporting |  |  |  |  |  | ✓ |  |  |  | ✓ |  | ✓ |
|  | Newborn indicators not captured in HMIS and reports | ✓ |  | ✓ | ✓ |  | ✓ | ✓ | ✓ | ✓ |  | ✓ | ✓ |
|  | Poor implementation of perinatal/clinical audits and reviews | ✓ |  |  |  | ✓ |  | ✓ | ✓ | ✓ |  | ✓ |  |
|  | Data quality is poor/limited |  |  |  |  | ✓ |  |  |  |  |  | ✓ | ✓ |
| **Community Ownership and Partnership** | Poor community and male involvement to facilitate care seeking for newborn health |  | ✓ | ✓ |  | ✓ |  | ✓ |  | ✓ |  | ✓ |  |
|  | Socio-cultural and gender barriers and challenges faced by mothers | ✓ |  | ✓ |  | ✓ | ✓ | ✓ | ✓ | ✓ | ✓ |  | ✓ |
|  | Access constraints (distance, cost of travel and care) | ✓ |  |  |  |  | ✓ | ✓ | ✓ | ✓ | ✓ |  | ✓ |
|  | Limited community awareness/ strategies to facilitate knowledge about newborn issues | ✓ | ✓ | ✓ |  |  | ✓ |  |  |  |  | ✓ | ✓ |
|  | Limited communication skills of health providers and lack of IEC materials in appropriate local languages |  |  | ✓ |  |  |  |  |  |  |  |  | ✓ |

DRC: Democratic Republic of Congo; HMIS: Health Management and Information Systems; IEC: Information Education and Communication

# B. Table S2: Bottlenecks for neonatal resuscitation

| **Health System Building blocks** | **Bottleneck Category** | **Africa** | | | | | | **Asia** | | | | | |
| --- | --- | --- | --- | --- | --- | --- | --- | --- | --- | --- | --- | --- | --- |
|  |  | Cameroon | DRC | Kenya | Malawi | Nigeria | Uganda | Afghanistan | Bangladesh | India | Nepal | Pakistan | Vietnam |
| **Leadership and Governance** | Guidelines unavailable, not updated, poorly disseminated/ implemented | ✓ | ✓ | ✓ | ✓ |  | ✓ | ✓ | ✓ | ✓ | ✓ | ✓ |  |
|  | Policy not available or updated | ✓ |  | ✓ | ✓ |  |  |  |  |  | ✓ | ✓ |  |
|  | Policy restriction on unskilled workers providing service but two thirds of deliveries attended by unskilled workers |  |  |  |  |  |  |  | ✓ |  |  |  |  |
|  | Most births occur at home attended by unskilled workers |  |  |  |  | ✓ |  |  |  |  |  |  |  |
|  | Lack of awareness and advocacy for leaders; Lack of partner-driven support |  |  |  |  |  |  |  |  |  |  |  | ✓ |
|  | Lack of coordination between Ministries of Health and Education |  |  |  |  |  | ✓ |  |  |  |  |  |  |
|  | Poor public-private partnership /private sector compliance to national standards |  |  |  |  |  |  |  | ✓ |  |  |  |  |
| **Health Financing** | Inadequate funding/ budget allocation | ✓ | ✓ | ✓ | ✓ | ✓ | ✓ | ✓ | ✓ |  | ✓ | ✓ | ✓ |
|  | Lack of awareness on financial guidelines |  |  |  |  |  |  |  |  | ✓ |  |  |  |
|  | Funding not specific /prioritised for resuscitation | ✓ |  |  | ✓ |  | ✓ |  |  |  |  |  |  |
|  | Poor coverage of health insurance |  |  |  |  | ✓ |  |  |  |  |  |  |  |
|  | High out-of pocket expenditures for maternal and newborn services |  |  |  | ✓ | ✓ |  |  |  |  |  | ✓ |  |
| **Health workforce** | Poor competency, skills and knowledge of providers | ✓ |  | ✓ |  | ✓ | ✓ | ✓ | ✓ |  | ✓ | ✓ | ✓ |
|  | Inadequate numbers and poor distribution of health workers | ✓ |  | ✓ |  | ✓ | ✓ | ✓ | ✓ |  |  | ✓ | ✓ |
|  | Inadequate in-service and pre-service training/ refresher courses | ✓ |  | ✓ |  |  | ✓ | ✓ |  | ✓ | ✓ | ✓ | ✓ |
|  | Poor supervision and mentorship |  | ✓ |  | ✓ | ✓ |  |  | ✓ | ✓ |  | ✓ |  |
|  | Lack of job description and job aids |  |  |  | ✓ | ✓ |  | ✓ |  |  |  | ✓ |  |
|  | Poor remuneration/ attitude of workers |  |  |  |  | ✓ |  | ✓ |  |  |  |  |  |
|  | Lack of/No training of community midwives |  |  |  |  |  |  | ✓ |  |  |  |  |  |
| **Essential Medical Products and Technologies** | Lack of supplies/equipment e.g. bag and mask not on essential equipment lists | ✓ | ✓ |  | ✓ | ✓ | ✓ | ✓ | ✓ |  |  | ✓ | ✓ |
|  | Inadequate procurement and logistics supply system |  |  | ✓ | ✓ | ✓ | ✓ | ✓ | ✓ | ✓ | ✓ | ✓ | ✓ |
|  | Poor standards / quality of supplied equipment |  |  | ✓ |  |  |  |  | ✓ | ✓ |  | ✓ | ✓ |
| **Health Service Delivery** | Ineffective/ weak referral mechanisms, lack of adequate transport for post-resuscitation referral | ✓ |  |  | ✓ | ✓ |  | ✓ |  | ✓ |  | ✓ | ✓ |
|  | Poor quality of care; poor adherence to standards for use of commodities; poor monitoring or quality assurance mechanisms | ✓ |  |  | ✓ | ✓ |  |  |  | ✓ |  | ✓ |  |
|  | Poor availability, maintenance and quality of equipment |  |  |  |  |  | ✓ | ✓ | ✓ |  |  | ✓ | ✓ |
|  | Poor coverage/ availability of service at district level | ✓ |  |  | ✓ |  |  |  |  |  |  | ✓ | ✓ |
|  | Inadequate number/skills of health workers providing resuscitation |  |  |  |  |  |  | ✓ |  | ✓ |  | ✓ |  |
|  | Ineffective communication between health facilities and the community | ✓ |  |  |  |  |  |  |  | ✓ |  |  | ✓ |
| **Health Information System** | Poor documentation of resuscitation service; poor implementation of perinatal death reviews and audits | ✓ |  | ✓ | ✓ | ✓ |  | ✓ |  | ✓ | ✓ | ✓ | ✓ |
|  | Newborn indicators not captured in HMIS | ✓ | ✓ |  | ✓ |  |  | ✓ | ✓ | ✓ |  | ✓ | ✓ |
|  | Data quality is poor/limited |  |  | ✓ |  | ✓ |  |  |  |  |  | ✓ | ✓ |
| **Community Ownership and Partnership** | Poor community/male involvement/mobilisation to facilitate care seeking |  |  | ✓ |  | ✓ |  | ✓ |  | ✓ |  | ✓ | ✓ |
|  | Socio-cultural and gender barriers and challenges |  |  |  | ✓ |  |  | ✓ |  | ✓ |  | ✓ |  |
|  | Lack of community awareness, information, and counselling about perinatal complications / resuscitation | ✓ |  | ✓ |  |  |  | ✓ | ✓ | ✓ |  | ✓ |  |
|  | Limited availability of education and communication materials in local languages |  | ✓ |  |  | ✓ |  | ✓ |  |  |  | ✓ | ✓ |
|  | Access constraints (distance, cost of travel and care) | ✓ |  |  | ✓ |  |  | ✓ |  | ✓ |  |  |  |

DRC: Democratic Republic of Congo; HMIS: Health Management and Information Systems; IEC: Information Education and Communication

# C. Table S3: Solutions for basic newborn care

| Basic Newborn Care | | | | | | |
| --- | --- | --- | --- | --- | --- | --- |
| Health System Building Block | Africa | | | | | |
|  | Cameroon | DRC | Kenya | Malawi | Nigeria | Uganda |
| Leadership and Governance | - Introduce all aspects of global PEC basic care for newborns in the document SRMNI strategy including the monitoring of N-only at the community level - Introducing said aspects in the curricula of schools and training schools | - Integration of new guidelines on umbilical cord care in the standards document - Extension of standards and guidelines on basic neonatal care at all levels of the health pyramid. | - Establishment of MNH scale - Implementation plan with a committee to monitor implementation - GOK to work closely with CSOs, CBOs, Implementing partners and BMS manufacturers | - No solutions proposed | - Advocacy to relevant stakeholders at the three tiers of government to ensure policy implementation - Carry out sensitisation activities | - No solutions proposed |
| Health Financing | - Advocacy for budget allocation for health of the newborn - Equipping FS specific materials for PEC N-Do - Support the financing of newborn care (kits, grant, free) | - Advocacy and social mobilisation for mutual health - Subsidies for newborn care by State. | - Using the RH-BP and the MNH scale up implementation for MNH resource mobilisation through mapping of all MNH partners - Advocacy for increased funding for MNH including the MNCH Bill - Advocacy Universal health care - MNH Implementation Plan established with close monitoring of its implementation at all levels. | - No solutions proposed | - Advocacy to government to increase funding for maternal and newborn care - Ensure due process is followed in procurement following adequate needs assessment - Increased involvement of relevant stakeholders in procurement decisions - Widen the scope of health insurance | - No solutions proposed |
| Health Workforce | - CHWs involved in the PEC n-do - (Revise training modules) - Extend training on PEC N-Do throughout the country | - Capacity building of health workers through skills-based approach (CBA) - Improved working conditions for staff. | - Advocate implementation of HRH strategy - Advocate for Counties to incorporate and implement HRH issues in their strategic plans - Scale up of “Heshima (Respect at Birth)” project which should be a component of the MNH scale up implementation Plan | - No solutions proposed | - Increase number of training institutions in deficient areas - Improved retention of HCWs through improved welfare packages - Task-shifting policy should be developed and implemented - Urgent updates of pre-service training curricula for all cadres of healthcare workers - In-service training and reorientation of health workers. | - Reanalyse and revise the staffing norms for facilities to ensure adequate numbers of midwives and nurses caring for newborns - Better HR management including job descriptions, performance appraisals - Involve district level Quality Improvement team in improving the cleanliness, appearance and general environment of health facilities - Develop and test a new bonus payment system for midwives based on number of deliveries and with documented post-natal visits - Revitalise the system for sanctions/rewards for health workers performance - Develop mentorship program for facility midwives and nurses to strengthen skills in newborn care and resuscitation - Midwifery pre-service training should include competency-based techniques for building skills, especially for newborn care |
| Essential Medical Products and Technologies | - Popularise the use of first-line chlorhexidine for cord care in health facilities (in place of the alcohol) and communities | - Advocacy for staffing of health facilities in equipment, materials and inputs for the newborn - Inclusion of 7.1% chlorhexidine on NEML - Supply of 7.1% chlorhexidine in the country. | - Include in NEML - Work with drug manufactures in country for availability of appropriate formulation and with PPP for importation of gel form | - No solutions proposed | - Expedite action on implementing the policy on use of chlorhexidine for cord care - Provision of essential medicines, commodities and equipment. | - Work with Uganda team on the UN Commission on commodities on logistics and supply chain management - Focus on district and health facility level supply management - Make sure that the Neonatalie kits are procured and distributed to facilities that are trained in neonatal resuscitation to maintain skills - Work with the MSH/SURE project on specific measures to improve supply chain management for newborn commodities and supplies |
| Health Service Delivery | - Advocacy and awareness of scholarly society, community leaders care about N-Do - Strengthen the capacity of health facility staff on care N-Ne including the continuum of care in post-natal | - No solutions proposed | - Establishment and monitoring of MNH scale up implementation Plan - Strengthen the already existing PPP unit within the Ministry of health and enhance collaboration with other ministries - MNH Scale Up Implementation Plan - To be addressed in the MNH Implementation Plan - Close monitoring of the MNH Implementation Plan | - No solutions proposed | - Multi-sectoral collaboration in solving problems of access e.g. provision of access roads, water and power supply, sanitation and infrastructure - More implementation of PPP. | - No solutions proposed |
| Health Information System | - Integrate information on the care of n-base is in the NHIS - Disseminate protocols for clinical audits and reviews of perinatal deaths of newborns. | - Integration of indicators on basic newborn care in the NHIS (under revision) | - Work with HMIS and DHIS to include NBC indicators - PNC to be a critical component of the MNH Implementation Plan | - No solutions proposed | - Expedite action on the use of updated HMIS tools which include the newborn indicators - Initiate and institutionalise clinical audit at all levels of healthcare | - Review and update maternity and post-natal registers - Revise HMIS to collect newborn indicators with clearer definitions and strengthen the use of the information by facilities during discussion at monthly meetings - Develop an e-Health system using cell phones for tracking and follow up on post natal care visits with VHTs - Strengthen the role of Health Assistants and Health Inspectors to link VHTs to the personnel at the health centres encouraging them to attend monthly meetings and encouraging health personnel to reach out to VHTs |
| Community Ownership and Partnership | - Integrate specific strategies (SA, SM) to facilitate the use of n-ing services in remote rural areas - Organise against the reference-reference between community / FS and FS FS - Form the CSA PEC N-Do - Advocacy and community awareness on the identified barriers. | - Strengthening the system of communication and dissemination of standards and guidelines for the community in all ZS | - To be covered in the MNH Scale up implementation Plan | - No solutions proposed | - Engaging communities and leaders in sensitisation fora e.g. town hall meetings and focus group discussions | - Develop knowledge of TBAs and/or VHTs on routine newborn care (drying and wrapping, immediate breastfeeding, delayed bathing, etc) to educate mothers - Develop and implement a comprehensive behaviour change communications programs (involving churchs, male involvement, mass media, etc.) - Train Health facility workers to counsel mothers on the importance and actions to be taken for post natal care |

| Basic Newborn Care | | | | | | |
| --- | --- | --- | --- | --- | --- | --- |
| Healthy System Building Block | Asia | | | | | |
|  | Afghanistan | Bangladesh | India - AP | India - Odisha | Nepal | Vietnam |
| Leadership and Governance | - No proposed solutions | - Revise job descriptions of basic health workers to include postnatal visits and essential (basic) newborn care - Introduce standardised accountability mechanism for PNC including essential (basic) newborn care both in public and private sectors | - All the training modules to be approved by one technical group to harmonise the content - Yearly review and updating with latest information - Regular dissemination through monthly meetings at district & block level - Need for development of guidelines for a holistic approach for feeding practices - Clear guidelines to be formulated and disseminated | - Guidelines should be regularly updated by a technical committee at the national level - Even private sectors and PSUs should use the protocols and report to the state. | - No solutions proposed | - Newborn care at home should be integrated into M&E plan of the DOH, with a scoring system - In disadvantageous areas: need to improve the remuneration policies for health - MoH to organise a separate advocacy workshop for leaders of health facilities on the importance of newborn care |
| Health Financing | - No proposed solutions | - Specific budget allocation for commodities, logistics related to PNC, basic newborn care needs to be ensured in the operational plan of both DGHS and DGFP | - The MOIC meeting at the district level to be utilised to disseminate financial guidelines and budgetary allocation and MOs in turn to be informed by the MOIC - MOIC meetings at the district level to be utilised for disseminating clear and uniform guidelines for transportation | - Timely fund flow requires timely approval of PIP from GoI and timely submission of UC from the districts - Buffer funds to be created | - No solutions proposed | - To mobilise fund for support newborn care for disadvantages families - To increase the public fund - To mobilise from UNs, donors - To increase IEC/BCC, prioritising areas with high morbidity and mortality |
| Health Workforce | - No proposed solutions | - Ensure training and refresher training of all basic health workers (public and private) on ENC and PNC - Ensure distribution and use of job-aids. | - Policy for yearly recruitment - Empowerment of the MOIC for administrative action against third party contractual staff - Review of the existing financial provisions and monitoring of timely payments - Training status mapping and regular updating of knowledge through hand holding and mentoring visits - Data entry operator to be utilised for overall data entry and not only Arogya-Shree | - Appropriate HR policy need to be introduced - Salary of SNCU staff nurses should be at par with government nurses - Selection of ASHAs should be appropriate as per the criteria - Performance of the ASHA needs to be evaluated every 3 years - HBNC training quality should be improved - part trainings to be accredited - Additional ANMs must be appointed at places where appropriate ASHAs is not available | - No solutions proposed | - DoH to have plan for training and allocate fund to various training types: training by request, short-term, long-term training - To develop standardised training program. Need to have separated certification for essential newborn care and newborn resuscitation - Health workers who already have received training on neonatology need to be deployed in right workplace, without reassignment - To strengthen training for health communicators on professional knowledge - To increase remuneration to health workers who work on newborn care, newborn resuscitation |
| Essential Medical Products and Technologies | - No proposed solutions | - Skill of the service providers on neonatal care to be ensured by providing capacity development/training and mentoring - Development of structured performance appraisal system at a regular intervals and dissemination of findings/feedback for improvement - Promote delivery by skilled birth attendant and facility delivery | - Knowledge of program managers about the existing standards need to be reinforced through: GO letters from state - Monthly meetings to be utilised for dissemination | - Awareness generation activities and hands-on training for using ODIMS should be done | - No solutions proposed | - To reconsider the bidding mechanism regarding medicines and equipment for newborns - To focus on the value of quality for care and treatment, rather than to pick up the cheap price - To include antiserum IgG into the list of essential drugs |
| Health Service Delivery | - No proposed solutions | - Include7.1% Chlorhexidine in NEML and market it by pharmaceutical companies including promotion of social marketing. | - Extend existing mechanism to ensure ENC in private sector - Accreditation process based on standard guidelines - Government training programs to be extended to private sector and made mandatory - Community awareness though IEC, involvement of local leaders, SHG groups - Existing mechanism needs to be streamlined through effective and regular supportive supervision and monitoring and by strengthening of training centres - Need to procure at least one fully functional ambulance at PHC level | - Adequate staff to be provided at all DPs - Monitoring tool needs to be developed - Child health committee should be made functional - Child health review needs to be regularised on a monthly basis - There should be some policy for private sector too - MCTS needs to be strengthened | - No solutions proposed | - To strengthen collaboration between Obstetrics and Paediatrics for newborn care - To strengthen M&E for the safe referral package for newborn - To assess the need to give training to the health workers - To mobilise support from local authorities |
| Health Information System | - No proposed solutions | - Incorporation of basic newborn related indicators in HMIS - Establish functioning system for death review. | - Identify specific indicators for ENCR and incorporated in HMIS through consultative meetings at state level - Situational analysis to be carried out by block and district with guidance from state - Reinforcement through action plan based on the findings of situational analysis - Institutionalise in public health system through involvement of local district administration and include in the agenda for monthly review meetings | - No solutions proposed | - No solutions proposed | - To set up surveillance and review systems for certain important indicators such as neonatal mortality - To give training on calculation of indicators on newborn care for persons in charge of statistics |
| Community Ownership and Partnership | - No proposed solutions | - BCC activities through IPC and counselling targeting the pregnant women and their families - Develop functioning referral mechanisms - Massive campaign using multiple channels for dissemination of information on importance of PNC and basic newborn care | - Recruitment of ASHAs to be streamlined through rational identification and placement mechanisms - Awareness generation through IEC, mass media campaigns - Replacement of wages - Introduction and implementation of alternative methods of transportation such as doli’s | - No solutions proposed | - No solutions proposed | - To compile IEC/BCC materials suitable for local circumstances, especially to the ethnic minorities |

| Basic Newborn Care | | | | | | |
| --- | --- | --- | --- | --- | --- | --- |
| Healthy System Building Block | Pakistan | | | | | |
|  | AJK | Baluchistan | Gilgit-Baltistan | Khayber Pakhtun | Punjab | Sindh |
| Leadership and Governance | - Measures to promote adherence to standards and clinical protocols on Basic newborn care are: Capacity building skilled birth attendants in competencies of immediate newborn care - Provision of checklists specifying key actions for the care and monitoring for newborn including warmth, hygiene, cord care, immediate exclusive breast feeding, counselling and support, support for alternative feeding methods, recognition of danger signs and care seeking timing of post natal visits - Monitoring for checking their competencies | - No solutions proposed | - No solutions proposed | - Basic Newborn care services should be made available through a need based and equitable geographic spread | - No solutions proposed | - No solutions proposed |
| Health Financing | - Appropriate allocation of funds for necessary equipment | - No solutions proposed | - Redistribution and allocation of funds | - The budget allocation should be made in such a way so that it addresses the coverage and quality issues surrounding Basic Newborn Care | - No solutions proposed | - No solutions proposed |
| Health Workforce | - Mechanism required for checking competencies of Health workers providing BEmOC services - Manuals detailing standards of practice to be provided to all staff. | - No solutions proposed | - Further training and accountability, monitoring | - Ensure that all service providers receive competency based pre-service and on-the-job training - The staff placements between urban and rural areas should be equitable and need based - Develop a strong Monitoring and supervisory system in line with the job description of the service providers | - No solutions proposed | - No solutions proposed |
| Essential Medical Products and Technologies | - Functional logistic system to assess and forecast the requirement of essential medicines and supplies for basic Newborn care | - No solutions proposed | - System for supplies and maintenance needs to develop | - Strengthen the Logistics Information System to make it more responsive to the logistical needs of all health facilities | - No solutions proposed | - No solutions proposed |
| Health Service Delivery | - Active efforts to be made by all health facilities to promote quality BNC Services. | - No solutions proposed | - No solutions proposed | - Ensure that staff is well trained on clinical guidelines and adheres to it - Develop a system for continuous quality improvement | - No solutions proposed | - No solutions proposed |
| Health Information System | - No solutions proposed | - No solutions proposed | - No solutions proposed | - Ensure that Basic Newborn care is part of the HMIS - Collect and analyse, and use data for quality improvement and informed decision making | - No solutions proposed | - No solutions proposed |
| Community Ownership and Partnership | - Awareness programs for community by CMWs and LHWs - Media campaign - IEC material | - No solutions proposed | - Awareness for girls, mother and females | - Raise awareness regarding Basic Newborn care and ensure community participation through culturally appropriate ways | - No solutions proposed | - No solutions proposed |

# D. Table S4: Solutions for neonatal resuscitation

| Neonatal Resuscitation | | | | | | |
| --- | --- | --- | --- | --- | --- | --- |
| Health System Building Block | Africa | | | | | |
|  | Cameroon | DRC | Kenya | Malawi | Nigeria | Uganda |
| Leadership and Governance | - Include part of neonatal resuscitation in SRMNI Plan and guide the IMCI | - Dissemination of standards and guidelines on basic neonatal care at all levels of the health pyramid | - Conduct a systematic review of related policies and strategies - Systematic address gaps identified - Include and regularly review pre-service training curricula - Include neonatal resuscitation in as a component of ENC at Level 2 facilities | - No solutions proposed | - Encourage ANC and delivery at hospital facilities - These should be adequately equipped and accessible | - No solutions proposed |
| Health Financing | - Advocacy for the mobilisation of funds for the care of the newborn, including resuscitation and strengthening of the public - private partnership | - Advocacy and social mobilisation for health insurance scheme | - Coordinated resource mobilisation efforts starting with a costed implementation plan, with prioritisation of neonatal HII, including neonatal resuscitation - PSM related financing should align with prioritised HII interventions - Advocacy for PSM to reflect the programme urgency with regards to neonatal HII including neonatal resuscitation | - No solutions proposed | - Advocacy at all levels for increased funding and resources - Health insurance for maternal and neonatal services | - No solutions proposed |
| Health Workforce | - Train health personnel in the care of the newborn, including resuscitation - Equipping MSDS (Material resuscitation of the newborn) | - Capacity building of health workers - Ensure the motivation of health personnel. | - No solutions proposed | - No solutions proposed | - Improve availability and placements of job aids at all levels - Training and retraining on neonatal resuscitation | - No solutions proposed |
| Essential Medical Products and Technologies | - Review the list of essential drugs and consumables to include the new one, included in the SYNAME | - Advocacy to increase public funds for the provision of equipment and inputs for the newborn | - No solutions proposed | - No solutions proposed | - Make wall charts, Ambu bags (all sizes) available - Strengthen LMIS for neonatal commodities and devices | - No solutions proposed |
| Health Service Delivery | - Organise the system of reference-reference - Promote the transfer in utero | - No solutions proposed | - No solutions proposed | - No solutions proposed | - Train and encourage the utilisation of checklists and SOPs - Collaboration between NGOs and partner activities with coordination by government | - No solutions proposed |
| Health Information System | - Include resuscitation data collection tools in routine | - Revision of the HMIS - Integration of indicators on basic newborn care in the HMIS | - No solutions proposed | - No solutions proposed | - Strengthen central, and national coordinated HMIS for neonatal resuscitation | - No solutions proposed |
| Community Ownership and Partnership | - Educate and involve communities and ASC in the PEC health problems of newborn | - Strengthening the system of communication and dissemination of standards and guidelines for the community in all health zones | - No solutions proposed | - No solutions proposed | - Widespread advocacy to stakeholders at the grassroots | - No solutions proposed |

| Neonatal Resuscitation | | | | | | |
| --- | --- | --- | --- | --- | --- | --- |
| Healthy System Building Block | Asia | | | | | |
|  | Afghanistan | Bangladesh | India - AP | India - Odisha | Nepal | Vietnam |
| Leadership and Governance | - No proposed solutions | - Initiatives to bring private sector under compliance. | - All the training module to be approved by one technical group to harmonise the content - Yearly review and updating with latest information - Regular dissemination through monthly meetings at district & block level - Need for development of guidelines for a holistic approach for feeding practices - Clear guidelines to be formulated and disseminated | - No solutions proposed | - No solutions proposed | - MoH to organise a separate advocacy workshop for leaders of health facilities on the importance of newborn care |
| Health Financing | - No proposed solutions | - Adequate fund allocation in OPs for organising need-based and refresher training and to procure and replace non-functional resuscitation devices | - The MOIC meeting at the district level to be utilised to disseminate financial guidelines and budgetary allocation and MOs in turn to be informed by the MOIC | - No solutions proposed | - No solutions proposed | - To mobilise fund for support newborn care for disadvantages families - To increase the public fund - To mobilise from UNs, donors |
| Health Workforce | - No proposed solutions | - No solutions proposed | - Policy for yearly recruitment - Empowerment of the MOIC for administrative action against third party contractual staff - Review of the existing financial provisions and monitoring of timely payments - Training status mapping and regular updating of knowledge through hand holding and mentoring visits - Data entry operator to be utilised for overall data entry and not only Arogya-Shree | - No solutions proposed | - No solutions proposed | - DoH to have plan for training and allocate fund to various training types: training by request, short-term, long-term training - To develop standardised training program. Need to have separated certification for essential newborn care and newborn resuscitation - To give training to midwives on essential newborn care and newborn resuscitation |
| Essential Medical Products and Technologies | - No proposed solutions | - No solutions proposed | - Knowledge of program managers about the existing standards need to be reinforced through: GO letters from state - Monthly meetings to be utilised for dissemination | - No solutions proposed | - No solutions proposed | - To reconsider the bidding mechanism regarding medicines and equipment for newborns to focus on the value of quality for care and treatment, rather than to pick up the cheap price |
| Health Service Delivery | - No proposed solutions | - No solutions proposed | - MOIC meetings at the district level to be utilised for disseminating clear and uniform guidelines for transportation - Extend existing mechanism to ensure ENC in private sector - Accreditation process based on standard guidelines - Government training programs to be extended to private sector and made mandatory - Existing mechanism needs to be streamlined through effective and regular supportive supervision and monitoring and by strengthening of training centres - Need to procure at least one fully functional ambulance at PHC level | - Monitoring tool needs to be developed - Child health committee should be made functional and manage the centres - HR at these centres should be made available | - No solutions proposed | - To strengthen M&E for coherence to the MoH’s decisions, technical guidelines - To assess the need - To give training to the health workers |
| Health Information System | - No proposed solutions | - No solutions proposed | - Specific indicators for ENCR need to be identified and incorporated in the HMIS through consultative meetings at state level - Situational analysis to be carried out by block and district with guidance from state - Reinforcement through action plan based on the findings of situational analysis - Institutionalise in public health system through involvement of local district administration and include in the agenda for monthly review meetings | - Real time data capturing mechanism needs to be established - CDR Mechanism with emphasis on neonatal component needs to be rolled out. | - No solutions proposed | - To set up surveillance and review systems for certain important indicators such as neonatal mortality, number of newborns in need of resuscitation |
| Community Ownership and Partnership | - Communication / advocacy through FHAG, mosque, school, building trust of community on health services, radio | - Use of job aids (simplified version) for service providers for counselling on community engagement to increase the use of emergency neonatal services - National awareness campaign on neonatal emergency and danger signs. | - Community awareness though IEC, mass media campaigns, involvement of local leaders, SHG groups - Recruitment of ASHAs to be streamlined through rational identification and placement mechanisms - Replacement of wages - Introduction and implementation of alternative methods of transportation such as doli’s | - Special BCC drive and convergence with education department needed in tribal areas - Peer to peer and change agent method of BCC should be adopted in these areas - Community representatives should be formally involved in audits. | - No solutions proposed | - No solutions proposed |

| Neonatal Resuscitation | | | | | | |
| --- | --- | --- | --- | --- | --- | --- |
| Healthy System Building Block | Pakistan | | | | | |
|  | AJK | Baluchistan | Gilgit-Baltistan | Khayber Pakhtun | Punjab | Sindh |
| Leadership and Governance | - No solutions proposed | - No solutions proposed | - Establishment of MCH wing at MoH level | - Develop and implement a clear policy on newborn resuscitations at all levels of care | - No solutions proposed | - No solutions proposed |
| Health Financing | - No solutions proposed | - No solutions proposed | - Priority setting and further budget allocation | - The budget allocation should be made in such a way so that it addresses the coverage and quality issues surrounding Basic Newborn Care | - No solutions proposed | - No solutions proposed |
| Health Workforce | - No solutions proposed | - No solutions proposed | - No solutions proposed | - No solutions proposed | - No solutions proposed | - No solutions proposed |
| Essential Medical Products and Technologies | - No solutions proposed | - No solutions proposed | - No solutions proposed | - No solutions proposed | - No solutions proposed | - No solutions proposed |
| Health Service Delivery | - No solutions proposed | - No solutions proposed | - No solutions proposed | - No solutions proposed | - No solutions proposed | - No solutions proposed |
| Health Information System | - No solutions proposed | - No solutions proposed | - No solutions proposed | - No solutions proposed | - No solutions proposed | - No solutions proposed |
| Community Ownership and Partnership | - No solutions proposed | - No solutions proposed | - No solutions proposed | - No solutions proposed | - No solutions proposed | - No solutions proposed |

# E. Figure S1: Subnational grading of bottlenecks for basic newborn care


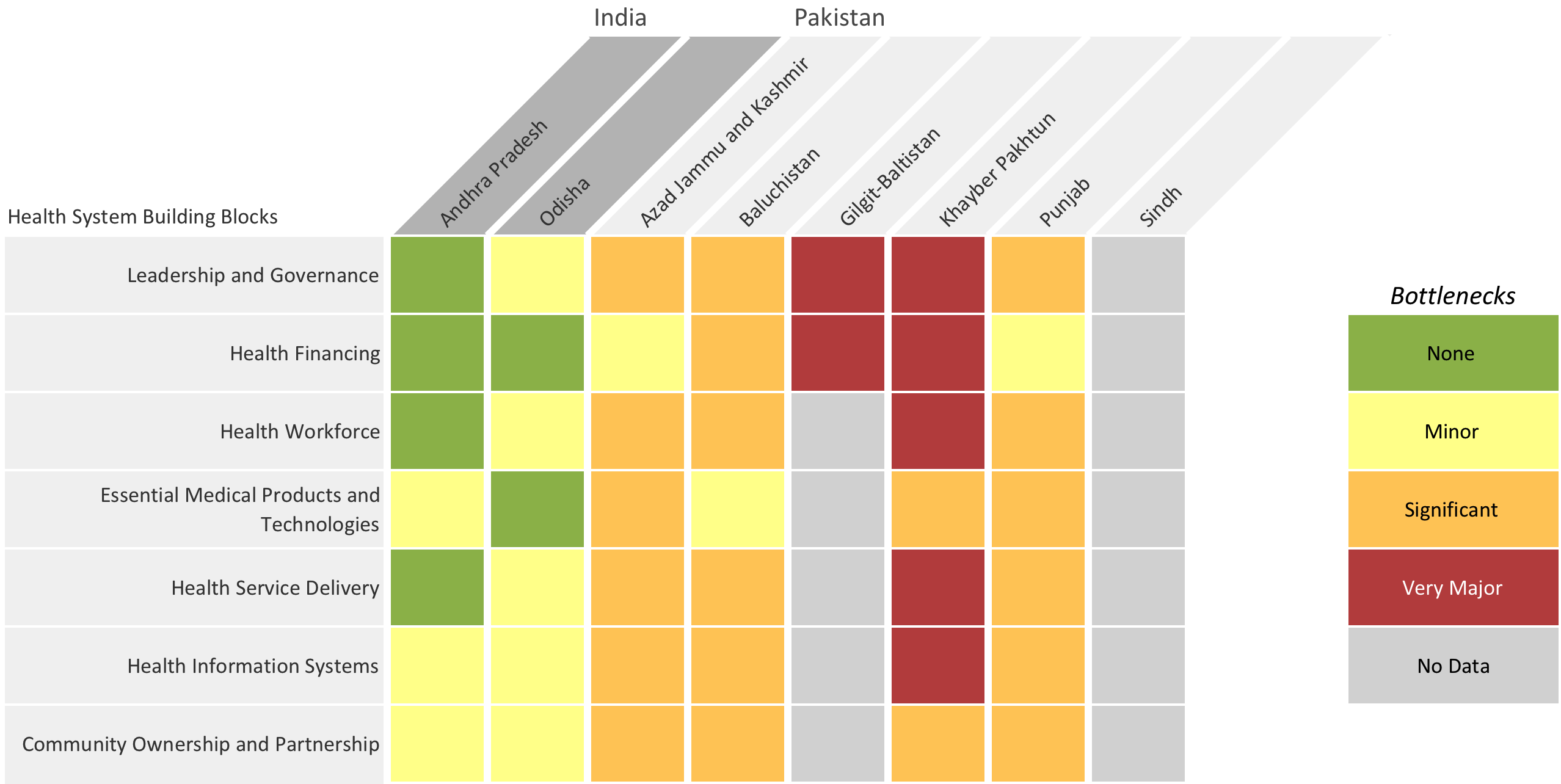


# F. Figure S2: Subnational grading of bottlenecks for neonatal resuscitation


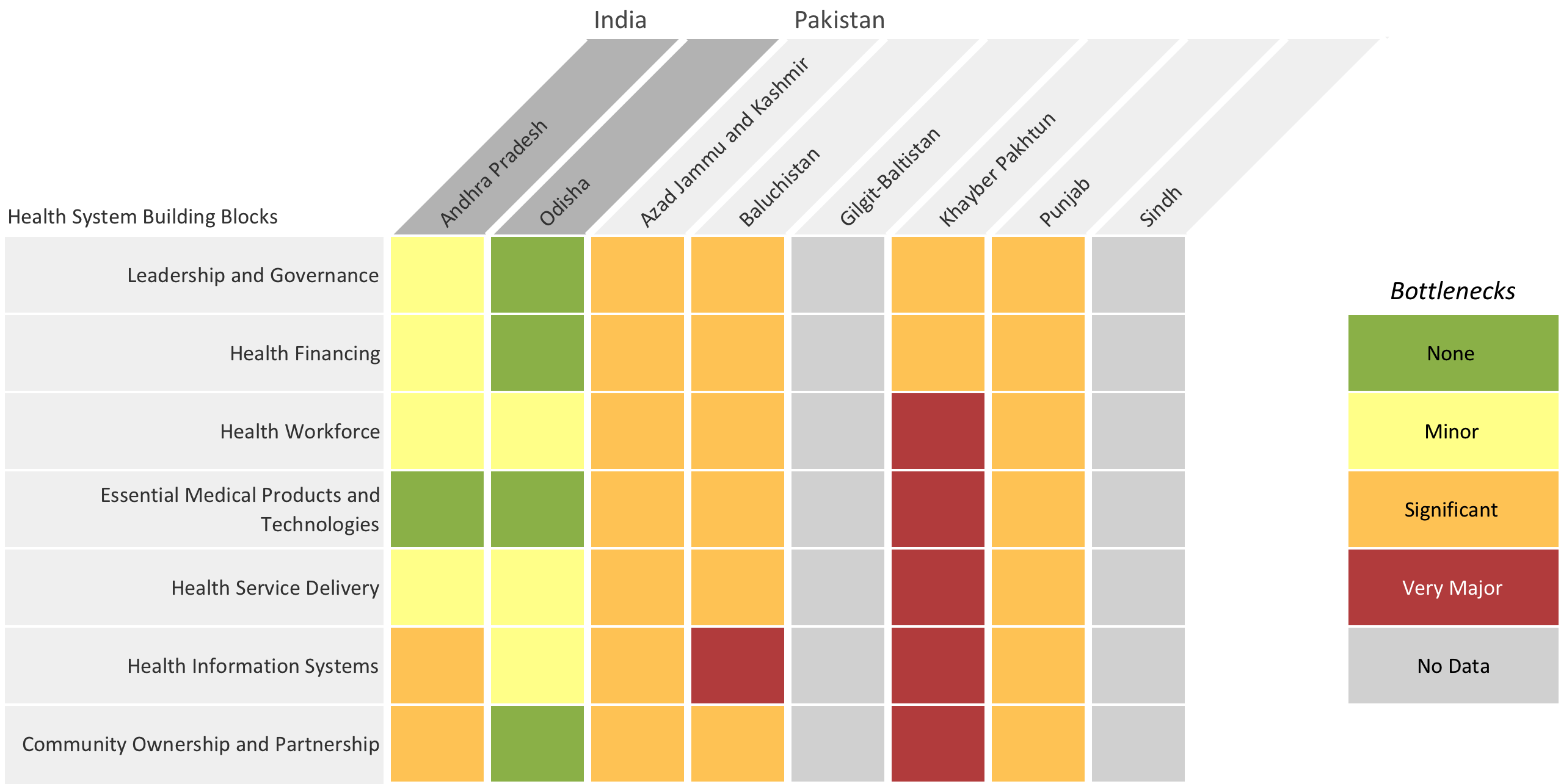


# G. Literature search strategy

**We used the following search terms in Pub Med, Cochrane Database of Systematic Reviews and Google Scholar. Appropriate limits were applied and only relevant articles (especially systematic reviews and meta-analysis) in English language were retrieved.**

**Newborn**

(neonate* OR newborn* OR new-born* OR neonatal / OR newborn, basic care/ OR newborn, essential care/ OR newborn, intrapartum care/ OR newborn, routine care/ OR newborn, care at birth/)

AND

**Resuscitation**

(bag and mask OR resuscitation, at birth OR resuscitation, intrapartum care OR resuscitation, labour room/ OR skills/ OR performance/ OR skills retention)

AND

**Immediate postnatal care**

(early newborn examination OR early newborn assessment OR care on first day of life OR care during first twenty four hours/ OR immediate postnatal care, hygienic practices/ OR immediate postnatal care, warmth or skin-to-skin or drying or wrapping or delayed bathing or radiant warmer / OR immediate postnatal care, cord care or umbilical cord care/ OR immediate postnatal care, nourishment or breastfeeding/)

AND

**Search terms for the discussion section include:**

**Leadership and governance**

(Leader or leadership) AND (political OR traditional OR religious/ OR advocacy OR engagement OR awareness OR commitment OR skills OR training)

Policy

Policy implementation

Guidelines and Standards

**Health Financing**

(Health) AND (financial access OR financial barrier OR out-of-pocket payment OR user fees OR financial plans OR hospital fees or costs)

Funding

Budget allocation

Social health insurance

National health insurance coverage

**Health workforce**

(Heath worker OR provider OR personnel) AND (training OR pre-service training OR in-service training OR refresher course OR competency OR skill OR performance OR attitude OR task-shifting OR retention OR job description OR job aids OR distribution OR incentives OR motivation)

Birth attendants

Health system personnel

Community health workers

Task shifting

Skills training

Competency based training

**Essential medical products and technologies**

(Equipment or supplies or commodities or medical technologies or essential drug list OR essential equipment list) AND (availability OR procurement OR quality OR hygiene OR maintenance OR use)

Bag and mask

Radiant warmers

Suction equipment

Chlorhexidine

Logistic systems

**Health service delivery**

(Health service or healthcare service or health service delivery) AND (quality of care OR standards OR adherence to standards OR guidelines and protocols OR hygienic practices OR birth practices OR newborn care)

Supervision

Mentorship

Evaluation and monitoring

Referrals and feedback

Outreach services

**Health information systems**

(Health information systems or Health management and information systems) AND (personnel OR tools OR skills or data analysis OR decision-making)

Newborn indicators

Monitoring tools

Perinatal audits

Clinical reviews

**Community:**

(Health) AND (information education communication OR community mobilisation OR utilisation OR sensitisation OR male involvement)

(Health) AND (education) AND (leaders OR women)

Community awareness

Community mobilisation

Care-seeking

Access to care

Male involvement

Information education and communication tools

Community and facility linkages

Community engagement
